# Targeting the gut–bone axis in osteoporosis: probiotic interventions and other microbiota-based therapeutic strategies

**DOI:** 10.1080/19490976.2026.2732528

**Published:** 2026-09-17

**Authors:** Kunyi Zhao, Zhishang Zhang, Zheng Tang, Xinai Man, Jiayi Liu, Ruiqi Feng, Kehao Li, Yaping Jiang, Tao Li

**Affiliations:** a Department of Joint Surgery, The Affiliated Hospital of Qingdao University, Qingdao, China; b Department of Oral Implantology, The Affiliated Hospital of Qingdao University, Qingdao, China; c School of Stomatology, Qingdao University, Qingdao, China

**Keywords:** Gut–bone axis, probiotics, osteoporosis, bone metabolism, microbiota-targeted therapies

## Abstract

Osteoporosis (OP) is a systemic bone metabolic disorder characterized by impaired bone homeostasis and increased fracture susceptibility. With the rapid aging of the global population, the number of fractures related to osteoporosis continues to increase, along with persistently high rates of disability and mortality, thereby imposing a huge and constantly growing burden on the healthcare system. Emerging evidence indicates that probiotics, as key regulators of host metabolic and immune networks, play critical roles in the pathogenesis, progression, and therapeutic modulation of OP. Through a bidirectional regulatory network known as the gut–bone axis, probiotics influence the dynamic balance between bone formation and bone resorption via multiple interconnected mechanisms, including immune modulation, microbial metabolite production, intestinal barrier integrity, and endocrine signaling. Recent animal and clinical studies further demonstrate that targeted modulation of the gut microbiota to restore gut–bone axis homeostasis can significantly improve bone mineral density and related skeletal parameters, highlighting the translational potential of probiotic-based interventions. This review systematically summarizes the molecular mechanisms by which probiotics regulate bone metabolism and places particular emphasis on emerging microbiota-based mechanisms and therapeutic strategies, with a specific focus on probiotics, aiming to provide a theoretical framework and future translational directions for the development of innovative microbiota-based treatments for OP.

## 1. Introduction

OP is a systemic skeletal disorder characterized by reduced bone mass, deterioration of bone microarchitecture, increased bone fragility, and elevated fracture risk. The World Health Organization defines it as a bone mineral density (BMD) 2.5 standard deviations or more below the average for healthy young adults of the same sex (T-score ≤ −2.5). Globally, approximately one-third of women and one-fifth of men over the age of 50 are affected, resulting in over 8.9 million fractures annually.^1^ It has become a major public health challenge in aging societies.^1–4^ OP is clinically silent in its early stages but progressively manifests as back pain, height loss, and kyphosis. A fragility fracture is its most severe consequence, commonly occurring in the vertebrae, hip, and distal radius. Hip fractures, in particular, carry a mortality rate as high as 20% and a disability rate exceeding 50%.^5^ At the cellular level, OP arises from a disruption of bone remodeling homeostasis, driven by excessive osteoclast (OC)-mediated bone resorption and/or insufficient osteoblast (OB)-mediated bone formation, highlighting the need for interventions targeting both arms of bone metabolism.

The gut microbiota is the most numerous, diverse, and mechanistically complex microbial community in the human body and is the most extensively studied microbiota associated with disease.^6^ The gut microbiota plays a crucial role in maintaining human health. Consequently, studies have reported its association with osteoarthritis (OA) and rheumatoid arthritis (RA).^7^ It may even serve as a reservoir for antibiotic resistance genes. Thus, the gut microbiota may emerge as a novel therapeutic target for treating OP and preventing related fractures. Moreover, significant individual variations exist in the gut microbiota across different people, influenced by several factors, including diet, age, genetics, exercise, and environment.^8^ Probiotics are primarily defined as live microorganisms that confer health benefits when administered in adequate amounts, while prebiotics are substances that promote the growth and/or beneficial metabolic activity of advantageous microorganisms. Several previous studies have explored how probiotic and prebiotic supplementation can influence bone metabolism by altering the state of the gut microbiota. These studies, conducted using various probiotic strains and experimental animal models of different diseases, confirm that the gut microbiota, as a key micro-ecosystem in the human body, is closely linked to human health.^9^ ^,^ ^10^

The “gut–bone axis” theory indicates that the gut microbiota is closely associated with the occurrence and development of OP. The immune response, metabolites, intestinal barrier function, and endocrine regulation are all critical factors influencing bone metabolism. Probiotics participate in regulating bone metabolic balance in OP through multiple mechanisms, such as modulating immune function, affecting nutrient absorption and metabolism, altering intestinal permeability, and influencing endocrine pathways. Current microbiota-targeted therapeutic strategies – including probiotic supplementation, dietary modifications, and fecal microbiota transplantation (FMT) – have been demonstrated to delay bone loss.^8^ In this review, we summarize the mechanisms of the gut‒bone axis and highlight microbiota-based therapeutic strategies for osteoporosis, with a particular emphasis on the application of probiotics, and highlights future therapeutic opportunities for preventing and treating OP.

## 2. Literature search strategy

Relevant literature was identified through searches of PubMed, Web of Science, and Scopus up to December 2025. The search strategy combined terms related to probiotics and bone metabolism, including “probiotics,” “gut microbiota,” “gut–bone axis,” “bone metabolism,” and “osteoporosis,” as well as specific cellular targets central to bone homeostasis, namely, “osteoclasts,” “osteoclastogenesis,” “osteoblasts,” and “osteogenesis.”

Original experimental studies, preclinical animal studies, and clinical studies that investigated the effects of probiotics or gut microbiota modulation on bone-related outcomes were considered. Mechanistic studies exploring immune, endocrine, or metabolic pathways linking the gut microbiota to bone homeostasis, particularly those detailing the direct or indirect regulation of osteoclast and osteoblast differentiation and activity, were also included.

Articles were excluded if they were conference abstracts, editorials, or studies not directly related to bone metabolism or osteoporosis. Only articles published in English were reviewed. In addition, reference lists of relevant articles were manually screened to identify additional studies of interest.

## 3. The mechanism by which probiotics treat OP through the gut‒bone axis

**Figure 1.**
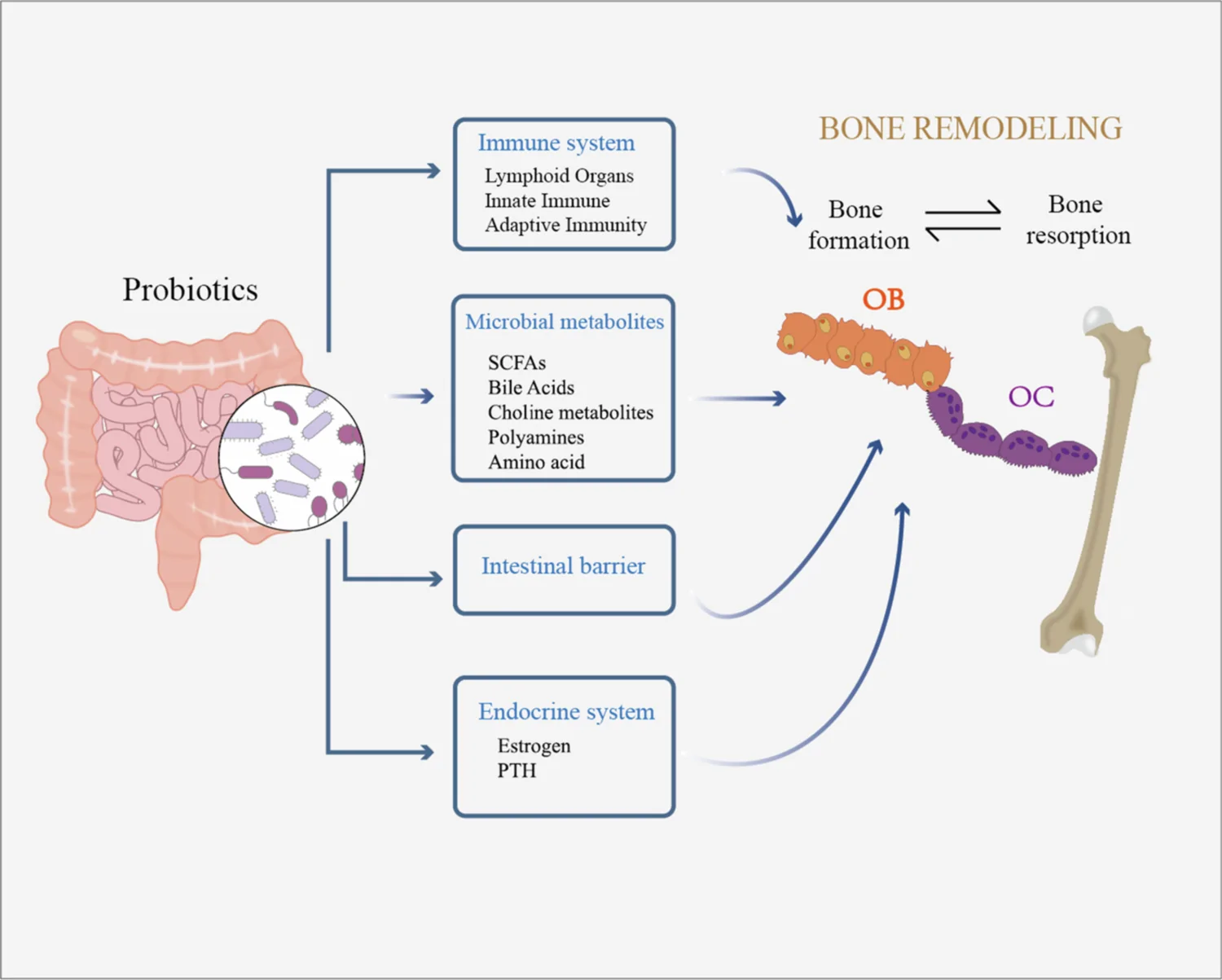
Regulation mechanisms of bone metabolism by Probiotics. The probiotics can regulate Treg and Th17 cells in the immune system; produce metabolites such as SCFAs, bile acids, choline metabolites, polyamines and amino acids; maintain the integrity of the intestinal barrier; and additionally regulate the metabolism of hormones such as estrogen and PTH, mediate the growth and differentiation of OB and OC, and participate in the dynamic balance between bone formation and resorption.

Through the bidirectional regulatory network termed the “gut‒bone axis,” the gut microbiota influences bone metabolic balance via multifaceted mechanisms, including: modulation of immune system function to suppress pro-inflammatory cytokine-mediated osteoclast activation; production of metabolites such as SCFAs and bile acids that directly participate in the dynamic equilibrium between bone formation and resorption; maintenance of intestinal barrier integrity to reduce chronic inflammation triggered by blood-borne pathogenic factors such as endotoxins; regulation of hormonal metabolic pathways involving estrogen and parathyroid hormone (PTH), which mediate osteoblast differentiation and mineralization processes (Figure 1).

### 3.1. Immunological mechanism

The symbiotic gut microbiota plays a multifaceted role in shaping host immune responses. Probiotics influence bone metabolism primarily by modulating the innate and adaptive immune systems, involving immune cells that reside both within the gastrointestinal (GI) tract and in systemic lymphoid organs. Here, we summarize how probiotics improve osteoporosis via immune cell-mediated mechanisms (Table 1).

#### 3.1.1. Lymphoid organs

DSS-induced bone marrow translocation of *E. faecalis* triggers trained immunity in bone marrow progenitors via the Mincle receptor, thereby enhancing the inflammatory response and improving subsequent anti-infection capacity. Importantly, this trained immunity in bone marrow progenitors is accompanied by functional alterations in the bone marrow microenvironment, including changes in hematopoietic lineage commitment that are closely linked to bone remodeling and osteoimmune regulation.^11^ Protopanaxadiol (PPD) prevents CTX-induced gut microbiota dysbiosis by increasing the relative abundance of *Lactobacillus*, *Oscillospiraceae*, *Turicibacter*, *Colidextribacter*, Lachnospiraceae, *Dubosiella*, and *Alloprevotella*, while reducing the abundance of Escherichia–Shigella.

Beyond restoring gut microbial balance, PPD promotes bone marrow hematopoiesis and modulates systemic immune responses that are directly associated with bone loss attenuation, including increasing splenic T lymphocyte numbers and regulating serum immunoglobulin and cytokine secretion. These immunological changes contribute to the suppression of CTX-induced bone loss and the preservation of bone mass by limiting excessive inflammation-driven osteoclast activation.^12^ FMT was performed from young donor mice to aged recipient mice. Single-cell RNA sequencing revealed that FMT from young mice upregulates the expression of FoxO signaling pathway-related genes in the hematopoietic stem cells of aged mice, thereby enhancing their lymphoid differentiation potential and suppressing the transcription of genes associated with inflammatory signaling pathways.

FMT remodeled the gut microbiota composition and metabolite landscape; Lachnospiraceae and tryptophan-related metabolites promoted hematopoietic recovery and rejuvenated aged HSCs. A significant increase in lymphoid differentiation and a decrease in myeloid differentiation were observed in aged recipient mice.^13^Long-term treatment with selected antibiotics that disrupted the gut microbiota was associated with reductions in CD20⁺ B-cell and CD3⁺ T-cell populations in the spleen. Alterations in the gut microbiome were associated with changes in bone mass and microstructure.^14^

#### 3.1.2. Innate immune system

##### 3.1.2.1. Macrophages

Macrophages dynamically regulate bone homeostasis through their M1 and M2 polarization states. A pro-inflammatory environment drives M1 polarization, and the inflammatory mediators they release, such as IL-1β and TNF-*α*, stimulate OC, exacerbate bone resorption, and lead to osteoporosis. Conversely, M2 macrophages promote bone formation and repair by secreting anti-inflammatory and osteogenic factors, such as IL-10, BMP-2, and TGF-β.^15^ The gut microbiota can regulate macrophages through their metabolites. Specifically, short-chain fatty acids, such as butyrate, markedly suppress the mRNA expression of genes associated with M1 macrophage polarization, including pro-inflammatory cytokines (TNF-*α* and IL-1β) and canonical M1 markers, while concurrently enhancing the transcription of M2 macrophage-associated genes, such as IL-10.^16^ Tryptophan derivatives, such as indole compounds, and melatonin can regulate the M1/M2 balance.^17^ In addition, with respect to direct regulation, acetate – a short-chain fatty acid produced by gut microbial fermentation – can directly act on mesenchymal stem cells/osteoblasts via activation of the G protein-coupled receptors GPR41 and GPR43, thereby inhibiting osteoblast differentiation and the expression of bone formation markers, while promoting adipogenic differentiation, ultimately leading to negative regulation of bone mass. Notably, no direct effect of this signaling axis on osteoclast differentiation or macrophage polarization was observed in this study.^18^ Meanwhile, lipoteichoic acid (LTA) present in the cell wall of probiotics (e.g., Bacillus subtilis, Enterococcus faecalis, and Lactobacillus species) also suppresses osteoclastogenesis, with the mechanism involving interference with osteoclast differentiation-related signaling (as demonstrated by downregulation of NFATc1 expression in the case of Staphylococcus aureus LTA).^19^ Specific probiotics or their metabolites can intervene in bone metabolism via the “gut‒bone” axis by targeting the functional state and activation pathways of macrophages. *Lactobacillus* rhamnosus LGG (LCM) inhibits RANKL-induced NF-κB activation in bone marrow-derived macrophages via the TLR6/NF-κB pathway, reducing OC and bone resorption. In OVX mice, it preserves trabecular bone structure, lowers serum *β*-CTx, and does not affect bone formation or liver/kidney function.^20^ *Bifidobacterium bifidum* FL228.1 and *Bifidobacterium animalis* F1-7 protect the intestinal mucosal barrier, inhibit intestinal inflammatory factors, and modulate the gut microbiota (the former increases the abundance of *Lactobacillus*, etc., the latter increases *Bifidobacterium* abundance and reduces *Desulfovibrio*, etc.), reduce M1-type macrophages, and inhibit excessive osteoclast generation.^21^ Prevotella histicola reduces the M1/M2 macrophage ratio in the intestines of titanium particle-treated mice, upregulates intestinal tight junction proteins and MUC2 to repair intestinal leakage, reduces inflammatory factors, downregulates CTX-1 and RANKL expression and the RANKL/OPG ratio, alleviates osteolysis, and its effect is closely related to the improvement of the gut microbiota.^22^ Gut microbiota-derived melatonin (MLT), although not a direct probiotic strain, can delay the progression of OP by modulating the gut microbiota (increasing the abundance of probiotics like Allobaculum and Parabacteroides), improving microbiota metabolism and increasing short-chain fatty acid production, regulating the M1/M2 macrophage dynamic balance, reducing serum levels of pro-inflammatory cytokines, and simultaneously restoring intestinal barrier function.^23^ Although numerous studies have shown that probiotics can partially inhibit bone loss by regulating macrophages, direct experimental evidence remains limited regarding how specific probiotic supplementation alters macrophage proportions and how the gut microbiota contributes to this process.

##### 3.1.2.2. Dendritic cells (DCs)

Dendritic cells play a complex dual role in osteoporosis. They can influence bone metabolism through various mechanisms, including participation in inflammation-mediated OC, activating T cells to regulate bone remodeling, transdifferentiating into osteoclasts under specific conditions, thereby forming a cycle that exacerbates bone destruction, and secreting TGF-*β* to exert anti-osteoporotic effects.^24^ DCs are the most potent and versatile professional antigen-presenting cells (APCs), capable of initiating adaptive immune responses and supporting innate immunity. DCs can be classified into plasmacytoid DCs (pDCs) and conventional DCs (cDCs), with cDCs further divided into two subsets, cDC1 and cDC2. DCs are highly effective antigen-presenting cells and are essential for the efficient activation of naive T cells. Although DCs play diverse roles in shaping T cell development, differentiation, and function, tolerogenic DCs (tDCs) primarily contribute to Treg differentiation and homeostasis.^25^ The short-chain fatty acids butyrate and propionate inhibit the activation of bone marrow-derived DCs (BMDC) by suppressing the LPS-mediated expression of co-stimulatory molecules (such as CD40) and production of cytokines (such as IL-6 and IL-12p40).^26^ On one hand, dendritic cells can transdifferentiate into osteoclasts in the presence of M-CSF and RANKL, and do so more efficiently than monocytes, representing a novel type of osteoclast precursor cell involved in bone metabolism.^27^ Simultaneously, activated DCs promote OC indirectly by activating T cells, particularly Th17 cells, leading to the secretion of IL-17 and TNF-*α*. Furthermore, DCs also exert protective effects; their cDC2 subset can produce OPG to competitively inhibit RANKL and secrete anti-resorptive molecules such as TGF-*β*, to mitigate bone destruction.^15^ TGF-*β* derived from dendritic cells activates the Smad2/3 signaling pathway in osteoblasts. Upon activation of the type I receptor, Smad2 or Smad3 undergoes C-terminal phosphorylation, associates with Smad4 to form a transcriptional complex, and translocates to the nucleus, where it binds to the Smad-binding element (SBE) in target gene promoters. This R-Smad–Smad4 complex drives transcriptional responses to TGF-*β* signaling, thereby regulating osteoblast proliferation and differentiation.^28^ To elucidate the heterogeneity of osteoclasts, Madel et al. (10.7554/eLife.82037) employed monocyte-derived mature osteoclasts (MN-OCLs) and dendritic cell-derived osteoclasts (DC-OCLs) as in vitro models for tolerogenic osteoclasts (t-OCLs) and inflammatory osteoclasts (i-OCLs), respectively. Transcriptomic profiling revealed substantial differences in gene expression signatures between the two subsets, with i-OCLs exhibiting enrichment of genes associated with immune responses and co-stimulatory signaling pathways, most notably the pattern recognition receptors Dectin-1, Tlr2, and Mincle. In vivo, oral administration of the probiotic yeast *Saccharomyces boulardii* (Sb) in ovariectomized (OVX) mice, a model of postmenopausal osteoporosis, significantly reduced the proportion of i-OCLs and ameliorated trabecular bone loss. Mechanistically, both Sb-conditioned medium (Sb-CM) and specific agonists of Dectin-1 and Mincle effectively blocked the differentiation of bone marrow-derived dendritic cells into i-OCLs *in vitro*. Moreover, the inhibitory effect of Sb-CM on i-OCL differentiation was abrogated upon neutralization of Dectin-1 or Mincle, indicating that the metabolic products of Sb act primarily through these two receptors to exert their regulatory function. Collectively, these findings provide important experimental evidence for the osteoprotective role of probiotics via the dendritic cell-mediated regulation of inflammatory osteoclast differentiation, and offer a novel perspective for therapeutic strategies that selectively target osteoclast subpopulations.

##### 3.1.2.3. Natural killer (NK) cells

NK cells play a key role in maintaining bone integrity under pathological conditions. Studies have shown that the probiotic AJ2 can activate NK cells in vitro, significantly enhancing their ability to secrete cytokines such as IFN-*γ*. IFN-*γ* binds to osteoclasts, blocks the RANKL signaling pathway, and inhibits bone resorption, providing a new strategy for addressing tumor-induced osteoporosis and bone loss, and for improving bone quality.^29^ At the direct action level, probiotic AJ2 can activate NK cells and other immune cells to secrete IFN-*γ* in vivo. In this study, elevated IFN-*γ* levels were significantly correlated with reduced osteoclast activity and improved bone mass, suggesting that IFN-*γ* exerts an inhibitory effect on bone resorption in this model. When periodontal health is maintained, in an infection environment with the periodontal pathogen *Aggregatibacter actinomycetemcomitans* (JP2), neutrophils communicate with NK cells, initiating NK cell activation and cytokine expression, promoting NK-protected bone anabolic signaling, controlling osteoclast differentiation, and preventing bone loss.^30^Studies using osteoclasts and probiotics to expand and enhance NK cells from young and elderly donors have shown that, compared with young donors, NK cells from elderly donors exhibit reduced surface receptor expression, cytotoxic function, and cytokine secretion. Concurrently, elderly donor OCs show reduced surface receptors, the level of their mRNA decreased, leading to a diminished capacity to recognize and activate osteoclasts.^31^ The level of OC-induced NK cell expansion and functional activation is significantly lower.^31^

#### 3.1.3. Adaptive immunity

##### 3.1.3.1. Th17 cells

Th17 cells play a critical role in OVX-induced bone loss. These cells stimulate OC and bone resorption by increasing the production of IL-17, RANKL, and TNF-*α*. Bone marrow (BM) Th17 TNF-α +  cells not only promote OC differentiation without exogenous osteoclastogenic factors but also stimulate BM mesenchymal stem cells to secrete chemokines (Mcp1, Mip1α, and RANKL) and recruit inflammatory monocytes to the BM, leading to increased bone resorption.^32^ Dysbiosis of the intestinal microbiota upregulates the RNA expression of chemokine genes, enhances osteoclastogenesis, and promotes the migration of Th17 cells to the bone marrow, thereby exacerbating bone metabolic disturbances.^33^ Studies in OVX mice demonstrated that OVX increased the migration of TNF-expressing Th17 cells from the gut to the BM in C57BL/6 mice with an increased frequency of intestinal Th17 cells. Furthermore, manipulating pathways governing lymphocyte migration and homing to the BM^34^ results indicate that the interaction between the gut microbiota and the immune system is linked to the effects of estrogen withdrawal on trabecular bone.^34^ A recent animal study suggests that Bifidobacterium longum subsp. longum LPL-RH might inhibit Th17 immune function in ovariectomized rats partly by regulating the gut microbiota (S24-7, Prevotella) and the gut barrier, which reduces LPS from directly stimulating Th17 cells through the TLR4/MyD88/NF-κB pathway. In addition, LPL-RH can suppress Th17 cells and their secretion of IL-17A, and may also inhibit osteoclast activity through RANKL and M-CSF, ultimately preventing bone loss.^35^

##### 3.1.3.2. Treg cells

**Figure 2.**
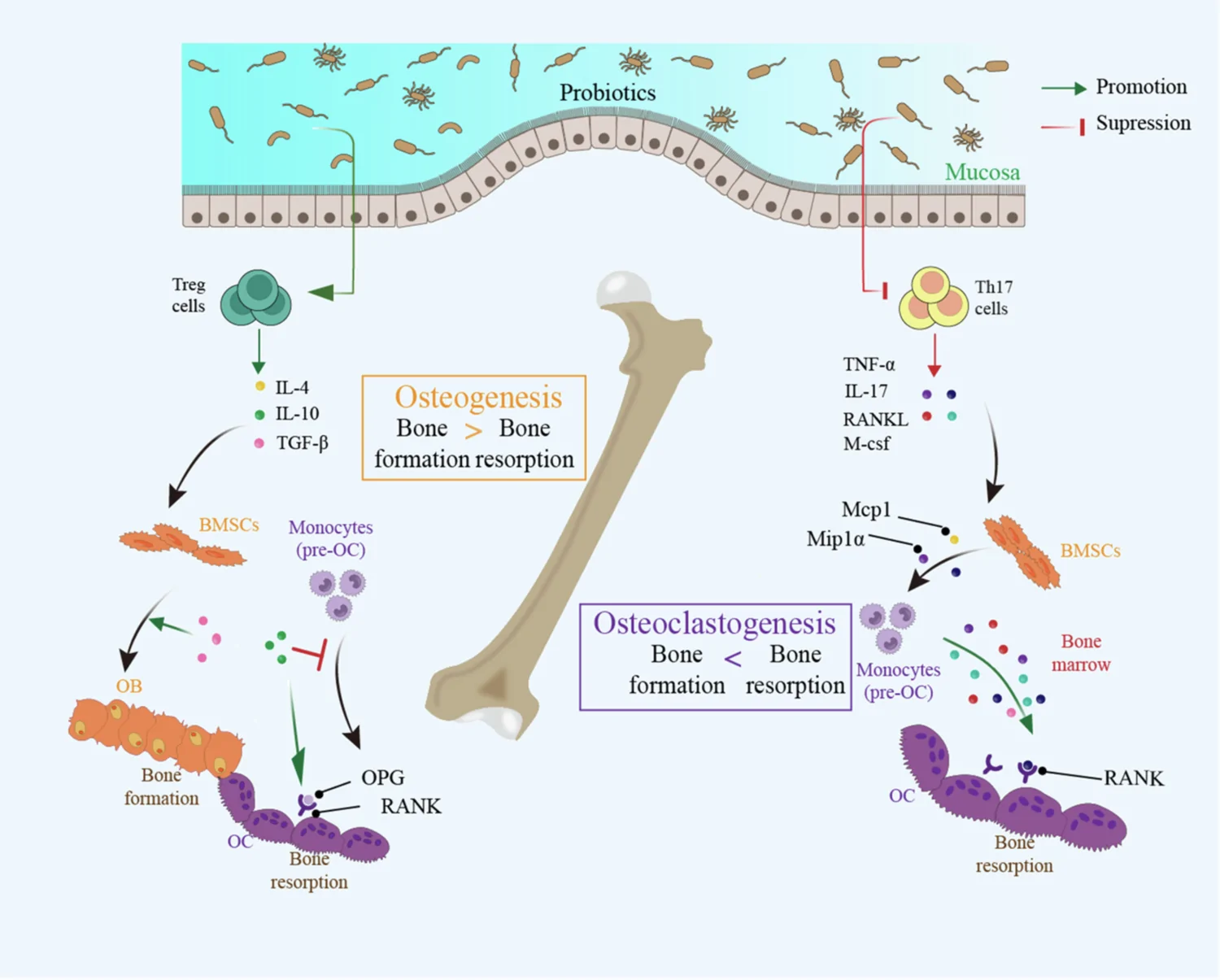
Probiotics regulate bone metabolism through the immune system. Th17 cells and Treg cells exhibit opposite roles in bone regulation. Probiotics influence the process of bone regulation by regulating the balance between Th17 and Treg cells. Treg cells, on the other hand, inhibit OC formation and promote OB by secreting anti-inflammatory cytokines such as IL-4, IL-10, and TGF-*β*. TGF-*β* promotes the proliferation and early differentiation of BMSCs, while IL-10 inhibits the differentiation and maturation of OC by upregulating the secretion of OPG. Th17 cells promote OC differentiation, leading to enhanced bone resorption; TNF-*α*-producing Th17 cells stimulate BMSCs to secrete chemokines, thereby increasing the recruitment of monocytes to the BM; and OC precursor cells then differentiate into the OC, resulting in bone resorption. The gut microbiota influences bone metabolism by inhibiting Th17 cells and enhancing Treg cells, thereby regulating immune factors, reducing inflammatory responses, and modulating the balance between OB and OC.

Treg cells, which are immunosuppressive CD4+ T cells,^36^ play a critical bone-protective role in osteoporosis by secreting anti-inflammatory cytokines to inhibit OC. Specifically, IL-10 downregulates RANKL and M-CSF expression while enhancing osteoprotegerin secretion, and it also stimulates CD8+ T cells to secrete Wnt10b, thereby activating the Wnt signaling pathway in osteoblasts and promoting bone formation.^9^ In the bone microenvironment, probiotic-induced Treg cells directly act on OC precursors and OB via the secretion of IL-10 and TGF-*β*, thereby suppressing OC differentiation and enhancing osteoblast function. Nevertheless, it is important to recognize that the differentiation, expansion, and migration of Treg cells to the bone marrow are themselves subject to regulation by systemic immune status and gut microbial signals; accordingly, this local direct effect cannot be dissociated from the systemic immune environment.^37^ Numerous studies have demonstrated that probiotics can prevent and treat bone loss by increasing Treg cell numbers and modulating the Th17/Treg balance. The gut microbiota influences regulatory T cell development through metabolites such as SCFAs, which promote CD4+ cell differentiation into Treg cells, elevate anti-inflammatory cytokine levels, and simultaneously inhibit Th17 differentiation and pro-inflammatory cytokine production.^38^ Certain microbes regulate bone remodeling by altering the Th17/Treg balance.^39^ In OVX rats, LGG treatment reversed the elevated TNF-*α* and IL-17 expression and reduced TGF-*β* and IL-10 expression in the colon and bone marrow. LGG treatment reversed these changes, significantly improved the Th17/Treg balance (decreasing Th17 and increasing Treg), and demonstrated protective effects against OVX-induced bone loss by enhancing the bone microstructure and biomechanics through the modulation of the gut and bone Th17/Treg balance, along with increased CTX-I and RANKL expression levels and reduced PINP and RORγt^40^ (Figure 2). The combination of *Butyricicoccus pullicaecorum* (Bp) and 3-hydroxyanthranilic acid (3-HAA) modulated the gut microbiota in ovariectomized rats, enriched relevant metabolic and immune pathways, reversed the increased Th17 cells and decreased Treg cells, restored Th17/Treg immune homeostasis, promoted osteogenesis, inhibited autophagy, and prevented PMOP^41^ In an ovariectomized postmenopausal mouse model, *Lactobacillus rhamnosus* (LR) inhibited osteoclastogenesis in vitro and regulated Treg-Th17 cell differentiation, while in vivo it reduced the proportion of osteoclastogenic CD4⁺Rorγt⁺ Th17 cells and increased anti-osteoclastogenic CD4⁺Foxp3⁺ and CD8⁺Foxp3⁺ Treg cells across different immunological sites, modulating related cytokines to alleviate bone loss.^42^ Bacillus clausii (BC) improves bone health by increasing Treg cells, reducing Th17 cells, regulating the Th17/Treg balance, decreasing pro-inflammatory cytokines, and elevating anti-inflammatory cytokine levels.^43^ Supplementing with Clostridium butyricum can help recover bone loss caused by segmented total abdominal irradiation by improving the shift of pro-inflammatory T cells in intestinal lymph nodes and the inflammatory microenvironment in the bone marrow. Specifically, this is shown by a decrease in Th17 cells and an increase in Treg cells in the gut, as well as a decrease in IL-17A and an increase in FOXP3 in the bone marrow. This suggests that *Clostridium butyricum* can regulate the gut immune balance, modulate the bone marrow immune environment, and prevent bone loss.^44^ Another study found that supplementing with LGG can boost Treg activation and reduce systemic inflammation, thereby alleviating bone loss caused by mechanical unloading. This effect might be related to gut microbiota regulation, restoration of SCFA levels, and improvement of gut barrier function.^45^

##### 3.1.3.3. B cells

In an ovariectomy (Ovx) mouse model, supplementation with *Bacillus coagulans* (BC) can ameliorate bone mineral density, bone strength, and bone microstructure by modulating the “gut–bone–immune” axis through the regulation of Bregs' anti-osteoclastogenic formation, immunosuppressive, and immunomodulatory potential. IL-10, primarily secreted by Tregs and Bregs, inhibits bone resorption, and BC administration significantly increases its serum level.^46^ *Bifidobacterium longum* (BL) inhibits the differentiation and functional activity of RANKL-induced OC in mouse bone marrow cells and human PBMCs. Its supplementation significantly enhanced bone mass and bone strength. BL stimulation increased the proportion of Bregs and efficiently induced anti-osteoclastogenic Tregs, while suppressing osteoclastogenic Th17 cells, indicating a key role for the “Breg-Treg-Th17” cell axis in alleviating Ovx-induced bone loss.^47^ Lactobacillus intervention significantly suppresses OC formation in ovariectomized mice, leading to an increase in trabecular number. Further studies showed that after *Lactobacillus* intervention, butyrate levels increased noticeably. Butyrate significantly inhibits B cell proliferation and RANKL expression on B cells, while also reducing RANK expression on OC to block the formation of the RANKL-RANK axis, thereby inhibiting OC differentiation and ultimately reducing bone loss.^48^ However, the direct inhibitory effect of butyrate on OC and its potential mechanism for suppressing RANKL on B cells are still unclear.

##### 3.1.3.4. CD8⁺ T cells

Osteoclasts can function as antigen-presenting cells, inducing the expression of Foxp3 and CD25 in CD8⁺ T cells, causing them to transform into regulatory CD8⁺ T cells (OC-iTcREG). TcREG cells, through their production of IFN-*γ*, inhibit OC and degrade TNFR-associated factor 6 in osteoclast precursors, blocking the required NF-κB signaling pathway, thereby limiting bone resorption and loss, and playing a key role in maintaining bone homeostasis.^49^ Another study found that receptor activator of NF-κB ligand (RANKL) has a dual function and participates in an important negative feedback loop in bone metabolism. High-dose RANKL directly stimulates osteoclasts and promotes bone resorption. In contrast, low-dose RANKL enhances the ability of osteoclasts to induce regulatory CD8 T cells via the Notch signaling pathway, thereby inhibiting bone resorption and even promoting bone formation.^50^ Tyagi et al. demonstrated that oral administration of *Lactobacillus rhamnosus* GG (LGG) significantly increased the trabecular bone volume in healthy mice, an effect primarily attributed to enhanced bone formation.^51^ Further mechanistic investigations revealed that LGG enriches the butyrate-producing gut microbiota and elevates circulating butyrate levels. Butyrate, in turn, expands the pool of regulatory T (Treg) cells in the bone marrow. These Treg cells interact with bone marrow CD8+  T cells, facilitating the assembly of the NFAT1-SMAD3 transcriptional complex, which subsequently upregulates the expression of the osteogenic Wnt ligand Wnt10b. This cascade ultimately promotes bone formation, likely through activation of the Wnt signaling pathway in osteoblasts. In this pathway, partial depletion of Treg cells in DEREG mice blocked the LGG/butyrate-induced increase in bone formation and the upregulation of Wnt10b mRNA in BM and CD8⁺ T cells; similarly, CD8⁺ T cell-specific knockout of Wnt10b abrogated these effects.^51^ Depletion of Treg cells or CD8+ T cell-specific ablation of Wnt10b abrogated these effects, underscoring the critical role of this axis in LGG-induced bone anabolism. Collectively, this study elucidates a potential mechanism by which probiotics promote bone formation via the “butyrate–Treg cell–CD8+ T cell–Wnt10b” axis, thereby providing novel experimental evidence and conceptual support for applying probiotics to prevent and treat osteoporosis.

**Table 1.** The role of gut microbiota in influencing bone metabolism through immune pathways.

Immune cell types	Crosstalk with the gut–bone axis	Ref.
Macrophages	*Lacticaseibacillus rhamnosus* LGG alleviates ovariectomy-induced bone loss in mice by suppressing osteoclastogenesis through TLR6/NF-κB regulation. Bifidobacterium bifidum FL228.1 and *Bifidobacterium animalis* F1-7 effectively inhibit bone loss by suppressing excessive osteoclast generation. *Prevotella histicola* reduces bone dissolution in mice by inhibiting osteoclast activity.	[44–46]
NK cells	AJ2 inhibits bone resorption by activating NK cells and enhancing IFN-*γ* secretion.	[51]
B cells	*Bacillus coagulans* (BC) can improve bone mineral density, bone strength, and bone microstructure by modulating the “gut–bone–immune” axis, increasing Bregs, IL-10, and anti-osteoclastogenic activity. *Bifidobacterium longum* (BL) inhibits RANKL-induced osteoclast differentiation and functional activity through the “Breg-Treg-Th17” cell axis, modulates Treg-Th17 cell balance, enhances IFN-*γ* and IL-10 levels while reducing IL-6, IL-17, and TNF-*α*, thereby alleviating bone loss.	[51,52]
CD8 + T cells	IFN-*γ* produced by CD8⁺ T cells inhibits osteoclastogenesis and degrades TNFR-associated factor 6 in osteoclast precursors, thereby blocking the required NF-κB signaling pathway and limiting bone resorption.	[53]
Th cells	The combined use of *Butyricicoccus pullicaecorum* (Bp) and 3-hydroxyanthranilic acid modulates the microbiota-Th17/Treg immune axis, promoting osteogenesis and inhibiting bone tissue autophagy.	[34]
Treg cells	The combination of *Butyricicoccus pullicaecorum* (Bp) and 3-hydroxyanthranilic acid (3-HAA) can prevent postmenopausal osteoporosis (PMOP) by promoting osteogenesis and inhibiting autophagy. *Lactobacillus rhamnosus* GG (LGG) ameliorates estrogen deficiency-induced osteoporosis by modulating the gut microbiome and intestinal barrier, as well as stimulating the Th17/Treg balance in both the gut and bone, thereby promoting osteogenesis. *Lactobacillus rhamnosus* (LR) alleviates bone loss by inhibiting osteoclastogenesis, regulating Treg-Th17 cell differentiation, and modulating related cytokines. *Bacillus clausii* (BC) inhibits bone loss, resulting in a net increase in bone mass.	[9,36,38,39]

### 3.2. Microbial metabolites

#### 3.2.1. SCFAs

**Figure 3.**
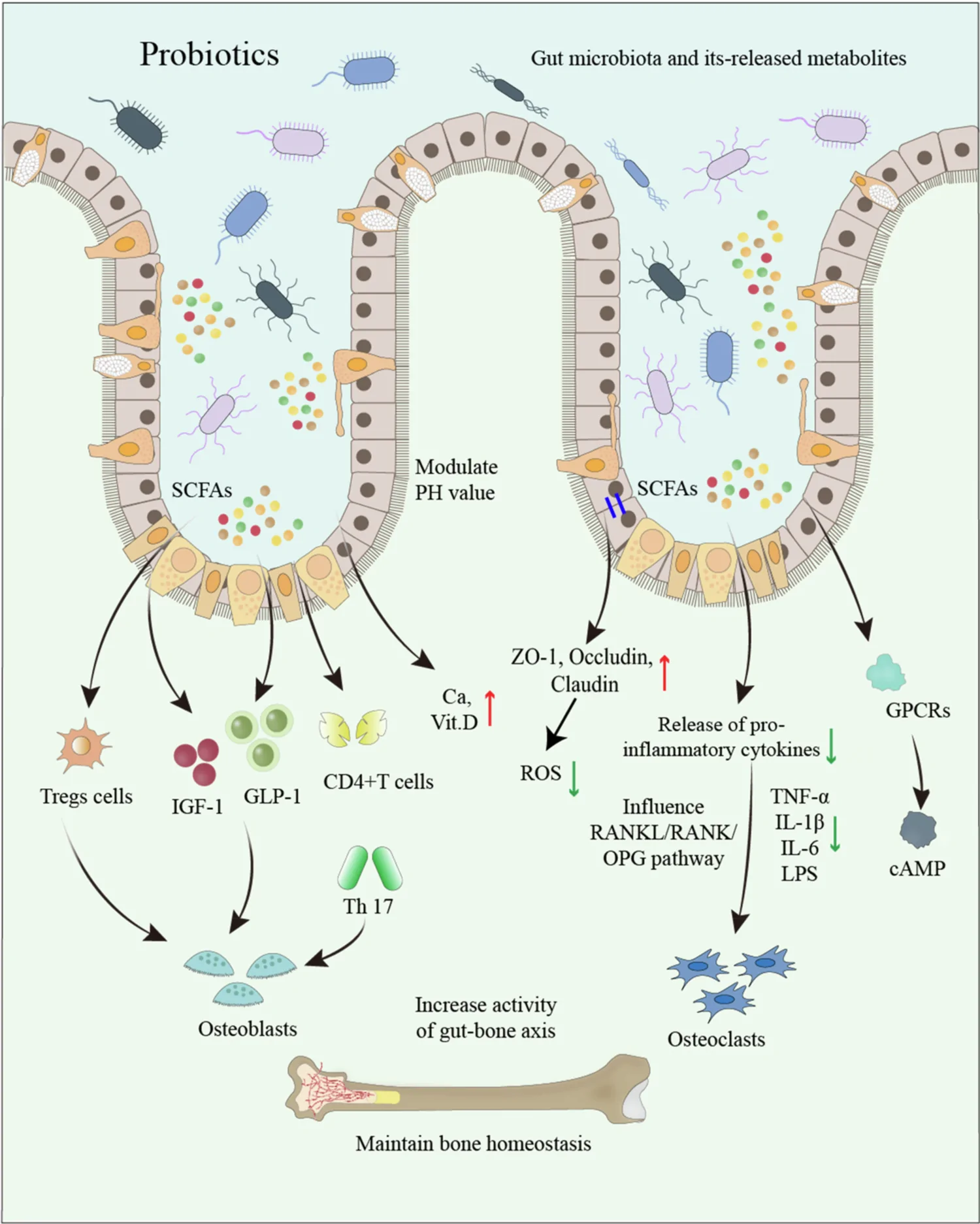
SCFAs affect bone metabolism processes through multiple mechanisms. Gut microbiota-derived SCFAs, such as butyrate and propionate, maintain bone homeostasis through several interconnected pathways: (1) directly inhibiting OC differentiation by promoting metabolic reprogramming and activating FFAR2; (2) promoting the expansion of Treg cells, which further stimulate CD8 + T cells to secrete the osteoanabolic Wnt ligand Wnt10b; (3) lowering intestinal pH and upregulating calcium-binding proteins to enhance intestinal Ca absorption; (4) increasing bone-derived IGF-1 secretion via the SCFA-IGF-1 axis to promote bone formation; and (5) stimulating the secretion of GLP-1, which bidirectionally stimulates osteoblast activity and inhibits bone resorption.

SCFAs constitute one of the most significant categories of gut microbial metabolites. Composed primarily of short-chain carboxylic acids, they are generated in the colon through microbial fermentation of indigestible carbohydrates, mainly dietary fiber. Major SCFAs include acetate, propionate, and butyrate. As the most extensively studied metabolites derived from the gut microbiota, SCFAs can diffuse from the gut into systemic circulation, where they play crucial regulatory roles in metabolic processes. SCFAs function as signaling molecules that mediate interactions between daily diet, the gut microbiota, and host health, exerting pivotal effects on immune, metabolic, and endocrine functions. Generally, probiotics mainly regulate the types and contents of fatty acids by altering the structure of the intestinal microbiota, thereby modulating bone metabolism and, to some extent, preventing OP. The targeted regulation of SCFAs may provide new approaches for treating bone-metabolism-related diseases and new targets for preventing OP. Furthermore, existing research evidence indicates that SCFAs may affect bone metabolism processes through multiple mechanisms (Figure 3).

##### 3.2.1.1. Direct inhibition of OC differentiation

Treatment with SCFAs or a high-fiber diet significantly increases bone mass and prevents both postmenopausal and inflammation-induced bone loss in mice. This protective effect correlates with the inhibition of osteoclast differentiation and bone resorption in vitro and in vivo, without affecting bone formation. Mechanistically, propionate (C3) and butyrate (C4) induce metabolic reprogramming in OC, enhancing glycolysis at the expense of oxidative phosphorylation, thereby downregulating essential osteoclastogenic genes such as TRAF6 and NFATc1.^53^ In a murine calvarial model of inflammatory osteolysis, C3 and C4 block CoCrMo particle-induced ASC speck formation and NLRP3 inflammasome oligomerization, inhibit particle-stimulated cleavage of caspase-1 and IL-1β, and suppress Gasdermin D-*N*-terminal fragment (GSDMD-NT) production to regulate pyroptosis. These SCFAs also suppress osteoclast differentiation and function by disrupting podosome patterning and actin ring formation.^52^ SCFAs primarily exert these effects through binding to G protein-coupled receptors, notably free fatty acid receptor 2 (FFAR2). Studies in FFAR2-deficient mice (FFAR2^−^/^−^) confirm that SCFA-mediated inhibition of OC depends on FFAR2 activation, independently of HDAC inhibition. FFAR2 deficiency exacerbates bone loss, while high-fiber diets (elevating SCFAs) or FFAR2 agonists (e.g., CTMB) effectively preserve bone mass, establishing FFAR2 targeting as a novel therapeutic direction for pathological osteolysis.^54^ Complementary human multi-omics analyses (metagenomics/targeted metabolomics/WGS) in Chinese perimenopausal/postmenopausal cohorts identified *Bacteroides vulgatus* as negatively correlating with BMD – a finding validated in a U.S. Caucasian cohort – and revealed that this bacterium impairs bone health by downregulating the microbiota-derived metabolite valeric acid, which positively correlates with BMD. OVX mice fed *B. vulgatus* exhibited increased bone resorption and impaired microstructure, whereas VA supplementation reduced resorption and improved microstructure. VA exerts these benefits by suppressing the pro-inflammatory factor RELA, enhancing the anti-inflammatory cytokine IL-10, inhibiting osteoclast-like cell maturation, and promoting osteoblast maturation in vitro. Thus, targeting *B. vulgatus* and supplementing VA represent promising strategies for OP prevention and therapy.^55^

##### 3.2.1.2. Promotion of Treg cells

SCFAs promote IL-22 production by CD^4+^ T cells and ILCs through GPR41 activation and HDAC inhibition. This mechanism involves upregulating AhR and HIF1α, which are regulated by mTOR/Stat3. HIF1α directly binds to the IL-22 promoter, and this binding is enhanced by SCFA-mediated histone modifications. SCFAs also induce Treg cell generation, improve their function, and enhance IL-22 production in human CD^4+^ T cells, collectively contributing to immune homeostasis.^56^ The binding of SCFAs to G protein-coupled receptors (GPCRs) on the cell surface may lower the level of intracellular cyclic AMP, activating immune responses, promoting Treg proliferation and differentiation, suppressing intestinal inflammation and osteoclast differentiation, and regulating bone metabolism.^57^ Additionally, Treg cells can stimulate CD^8+^ T cells to secrete the Wnt ligand Wnt10b. Wnt ligands stimulate bone formation by activating Wnt signaling in OB. Supplementation with *Lactobacillus rhamnosus* GG (LGG) can increase bone formation in mice. Following LGG intake or direct butyrate feeding to germ-free mice, Treg cells expand in the gut and bone marrow. Treg cells promote the assembly of the NFAT1-SMAD3 transcriptional complex in CD^8+^ T cells, thereby driving Wnt10b expression. The interaction between BM CD^8+^ T cells and Treg cells leads to the secretion of Wnt10b, an osteoanabolic Wnt ligand.^58^ Although SCFAs are typically anti-inflammatory, fermentable dietary fiber (via SCFAs) significantly improves survival in influenza-infected mice. A high-fiber diet alters BM hematopoiesis, increasing the number of specific macrophages with limited CXCL1 production, which reduces neutrophil recruitment to the airways and limits tissue immunopathology. Simultaneously, SCFAs enhance CD^8+^ T cell effector function by boosting cellular metabolism and promoting viral clearance. SCFAs establish immune balance through these dual complementary mechanisms, facilitating infection resolution while preventing excessive immunopathology.^59^ Jian-Gu Granules (Quercetin) restore gut microbiota richness, increasing beneficial bacteria *Lactobacillus, Blautia,* Prevotellaceae, and reducing potentially pathogenic bacteria *Desulfovibrio, Erysipelatoclostridium, Romboutsia*, and Butyricicoccaceae. This microbiota modulation increases SCFAs levels and decreases colonic epithelial permeability. Concurrently, it elevates the proportion of splenic Treg cells and reduces the levels of the osteoimmunology-related pro-inflammatory cytokines LPS, IL-1β, and TNF-*α*. These effects synergistically prevent bone loss and enhance bone strength.

##### 3.2.1.3. Promotion of Ca absorption

Butyrate, a major component of SCFAs, can directly inhibit OC differentiation in vitro and promote the differentiation of naïve T cells into Treg cells, which further induces OB differentiation. Regarding Ca absorption, SCFAs can enhance mineral solubility by lowering the pH in the intestinal lumen, thereby making Ca more readily absorbed by the body.^60^ ^,^ ^61^ SCFAs can upregulate the mRNA level of the calcium-binding protein D9k (i.e., Calbindin-D9k) in intestinal epithelial cells and enhance the calcium transport capacity.^62^ Simultaneously, SCFAs can directly increase the villus architecture and intestinal epithelial surface area. This leads to enhanced Ca absorption via the paracellular pathway and increased expression of calcium-binding proteins, thereby promoting Ca absorption in the intestinal villi.^63^

##### 3.2.1.4. Indirect regulation via modulation of IGF-1

Previous studies demonstrate that Insulin-like Growth Factor-I/Insulin-like Growth Factor-I Receptor (IGF-1/IGF-1R) signaling can be regulated by probiotics through increased production of their metabolic products, SCFAs.^64^ Local application of a high dose (80 μg/kg) of IGF-1 enhances mandibular growth and increases condylar bone mass and quantity in growing rats. These findings have implications for modulating mandibular growth and potentially enhancing condylar bone health and integrity.^65^ AMPKα1 is a key regulator of mesenchymal stem cell (MSC) senescence and differentiation. Studies using a phospho-mutant mouse model revealed that constitutively active AMPKα1 protects against age-related bone loss and promotes the osteogenic commitment of MSCs by enhancing bone-derived IGF-1 secretion. Mechanistically, this AMPKα1-mediated upregulation of IGF-1 signaling depends on CREB (cAMP response element-binding protein)-mediated transcriptional regulation. These findings elucidate the molecular basis for the protective role of AMPKα1 against skeletal aging via bone-derived IGF-1 secretion.^66^ *Bifidobacterium longum* subsp. *infantis* is a prevalent member of the gut microbiota in breastfed infants. The strain *Bifidobacterium longum* subsp. *infantis* CCFM1269 promotes skeletal formation in growing mice by modulating the gut microbiota composition and metabolites, and by regulating bone formation through the GH/IGF axis via the PI3K/AKT pathway.^67^ Colonization of germ-free (GF) mice with specific pathogen-free (SPF) gut microbiota initially increases both bone formation and resorption. While colonization in adult mice acutely reduces bone mass, long-term colonization promotes bone growth (longitudinal and radial), ultimately equilibrating bone mass. This process is accompanied by a significant increase in serum IGF-1, derived primarily from the liver and adipose tissue. Conversely, antibiotic depletion of the microbiota in conventional mice reduces serum IGF-1 and suppresses bone formation. Supplementation with the microbial metabolite SCFAs restores IGF-1 levels and bone mass, confirming that the gut microbiota stimulates bone anabolism via the SCFA-IGF-1 axis.^68^

##### 3.2.1.5. Promotion of GLP-1

SCFAs trigger glucagon-like peptide-1 (GLP-1) secretion in mixed colonic cultures in vitro.^69^ In intestinal epithelial cells, SCFAs primarily upregulate GLP-1 expression by activating the extracellular signal-regulated kinase (ERK) and p38 mitogen-activated protein kinase (p38 MAPK) phosphorylation pathways.^70^ Multiple studies have provided evidence for GLP-1's role in maintaining bone homeostasis. GLP-1 receptors have been detected in human osteoblastic MG63 cells, immature osteoblastic TE-85 cells, and the murine osteoblast-like MC3T3-E1 cell line.^71^ ^,^ ^72^ In TE-85 osteoblasts, GLP-1 activates the expression of c-Fos, a key gene regulating osteocyte proliferation and differentiation.^73^ GLP-1R knockout mice exhibit reduced bone mass and strength, decreased cortical area, and impaired collagen maturity.^74^ ^,^ ^75^ Administration of GLP-1 significantly upregulates OPG and osteocalcin (OCN) levels while improving trabecular microarchitecture in rats under physiological conditions, insulin resistance, and type 2 diabetes mellitus (T2DM).^76^ ^,^ ^77^ In hyperlipidemic OP rats, short-term GLP-1 or exendin-4 treatment restores bone mass and quality, accompanied by elevated OPG/OCN.^78^ OVX mice and liraglutide (a GLP-1 receptor agonist, GLP-1RA)-treated rats show significant improvements in trabecular number, volume, thickness, and areal BMD compared to controls.^79^ The core mechanism involves bidirectional regulation—stimulating osteoblast activity and inhibiting OC.^80^ Clinical evidence confirms the protective effect of GLP-1RAs on bone health in T2DM patients.^80^ Subcutaneous GLP-2 injection significantly reduces the bone resorption marker CTX in healthy postmenopausal women,^81^ ^,^ ^82^ highlighting the therapeutic potential of incretins (GLP-1/GLP-2) in regulating bone formation-resorption balance for metabolic bone diseases and postmenopausal OP. As a gut hormone, GLP-1 is closely linked to the gut microbiota and bone metabolism.^83^ Consumption of the probiotic VSL#3 promotes GLP-1 secretion and increases fecal butyrate levels in mice.^84^ The anti-diabetic effect of a 14-strain probiotic mixture in db/db mice appears to be associated with increased SCFA-producing bacteria, improved gut barrier function, and upregulated GLP-1 production.^85^ *Bacteroides* and *Lactobacillus* produce SCFAs, which activate GPR41/43 to stimulate GLP-1 secretion.^86^ Two independent cohort studies revealed dysregulated microbial bile acid metabolism in OA patients, characterized by reduced glycoursodeoxycholic acid (GUDCA) and a lower abundance of Clostridium species producing its precursor UDCA. Mechanistically, GUDCA inhibits the intestinal farnesoid X receptor (FXR), promoting GLP-1 secretion and alleviating OA in mice; GLP-1 receptor activation mimics this protection. Supplementation with C. bolteae or the FDA-approved drug UDCA mitigates OA via the gut FXR-GLP-1 axis, with human data indicating a reduced risk of joint replacement surgery with UDCA use. This finding reveals the “microbiota-GUDCA-gut FXR-GLP-1-joint” pathway as a novel therapeutic target for OA and OP.^87^

#### 3.2.2. Bile acids

Bile acids (BAs) are biosynthesized in the liver through cholesterol oxidation catalyzed by cytochrome P450 (CYP) enzymes, with their production modulated by the gut microbiota via the regulation of cholesterol-metabolizing enzymes.^88^ As terminal products of cholesterol catabolism, BAs exist primarily as primary and secondary forms in humans, playing essential roles in dietary lipid solubilization. Critically, primary BAs form bile salts in the liver that are secreted into the small intestine, where the gut microbiota metabolizes them into secondary BAs; this microbial modification alters both the quantity and profile of primary BAs, generating distinct metabolic effects relevant to skeletal health. Supporting this bone-BA connection, a cross-sectional study of 150 postmenopausal Chinese women revealed significantly lower serum total BA levels in the OP/osteopenia groups versus healthy controls, alongside positive correlations between serum BA levels and BMD at the lumbar spine, femoral neck, and total hip sites, and negative correlations with the bone resorption marker *β*-CTX.^89^ These findings collectively demonstrate that elevated serum BAs are associated with enhanced BMD and suppressed bone resorption, underscoring their pivotal role in bone metabolism.

##### 3.2.2.1. Promotion of OB cells

BAs stimulate osteoblast proliferation and differentiation by activating the FXR and TGR5 receptors. FXR activation enhances Runx2 expression to promote BMP-2-induced differentiation of BM mesenchymal stem cells (BMMSCs) into OBs, while FXR agonists simultaneously inhibit OC differentiation from BMSCs – an intervention that improves bone formation, reduces resorption, and restores bone homeostasis to alleviate OP.^90^ ^,^ ^91^ Specific BA derivatives modulate bone metabolism: the dual-receptor agonist SH-479 activates both TGR5 and FXR, more effectively suppressing OC while promoting osteogenesis and preventing bone loss in mouse models.^90^ Hydrophilic BAs (UDCA and its taurine-conjugated form TUDCA) exert antioxidant/anti-inflammatory effects, enhancing osteogenic differentiation of mesenchymal stem cells through Runx2/Osx upregulation, suppressing adipogenesis, and boosting OB viability and anti-apoptotic capacity in OVX-induced OP models.^92^ Notably, lithocholic acid (LCA) impairs osteocyte viability, promotes apoptosis, and elevates the RANKL/OPG ratio to stimulate bone resorption – effects effectively counteracted by UDCA. The gut microbiota critically regulates BA metabolism: *Prevotella copri* increases UDCA while reducing *Akkermansia* abundance in obese mice,^93^ and probiotic interventions (e.g., multispecies probiotics elevating unconjugated BAs^94^; *B. longum* SPM1207 enhancing fecal BA excretion^95^), indirectly modulate bone metabolism via BA dissociation/excretion kinetics. In OA patients, reduced Clostridium bolteae (a UDCA-producer) and its metabolite GUDCA are restored by C. bolteae supplementation or UDCA administration, alleviating OA via intestinal FXR inhibition and GLP-1 activation, with human data confirming reduced joint replacement risk.^87^ Given UDCA’s dual osteoanabolic/anti-resorptive actions in OP, targeting the microbiota-BA-gut axis offers a novel therapeutic strategy for OP/OA comorbidity. Collectively, modulating BA receptors (TGR5/FXR) or utilizing protective BAs (UDCA/TUDCA) to balance bone formation and resorption represents a promising OP treatment paradigm.

##### 3.2.2.2. Increase in vitamin D

Secondary bile acids serve as ligands for the vitamin D receptor, modulating the metabolism of 1,25-dihydroxyvitamin D₃.^96^ This regulatory function is further demonstrated by *Lactobacillus reuteri* NCIMB 30242, which increased serum 25-hydroxyvitamin D by 14.9 nmol/L (25.5%) during intervention.^97^ Collectively, these mechanisms contribute to the regulation of bone homeostasis and mineral balance.

##### 3.2.2.3. Regulation of immunity

Secondary bile acids derived from the gut microbiota suppress TNF-*α*-induced immune responses by inhibiting inflammasome expression and modulating ROS production and inflammatory reactions.^98^ Additionally, bile acid-related metabolites – including 3-oxolithocholic acid (3-oxoLCA) and iso-lithocholic acid (isoLCA) – regulate bone metabolism by influencing immune cell differentiation: 3-oxoLCA directly binds to the key transcription factor retinoic acid receptor-related orphan receptor gamma t (RORγt) to inhibit Th17 cell differentiation, while isoLCA promotes mitochondrial ROS production to modulate Treg differentiation.^99^

##### 3.2.2.4. Promotion of vascular regeneration

Angiogenesis is a critical process in bone regeneration. TUDCA (tauroursodeoxycholic acid), produced by the gut microbiota, promotes the cell migration and tubulogenesis of fibroblast activation protein (FAP)-induced endothelial progenitor cells (EPCs). This occurs through TUDCA’s modulation of the AKT and eNOS signaling pathways, which are associated with ERK 1/2 and JNK activation, ultimately accelerating neovascularization and bone formation.^100^ In summary, the gut microbiota alleviates OP by modulating bile acid metabolism, which subsequently influences OB function, vitamin D activity, immune responses, and angiogenesis.

#### 3.2.3. Choline metabolites

Choline metabolites, specifically L-carnitine and choline derived from high-fat dietary sources such as meat, fish, and dairy, are metabolized by the gut microbiota into trimethylamine (TMA), which is subsequently oxidized in the liver by flavin monooxygenases to form trimethylamine *N*-oxide (TMAO).^101^ While a population-based dietary intervention study suggested that circulating gut microbiota-derived TMAO might help prevent BMD loss during weight loss, with dietary fat potentially modifying the relationship between plasma L-carnitine changes and BMD changes,^102^ clinical evidence demonstrates that elevated serum TMAO levels are significantly higher in postmenopausal women with hip fractures compared to those with concurrent upper limb fractures. After adjusting for risk factors, high TMAO levels serve as an independent predictor for both isolated hip fractures and combined hip-upper limb fractures, indicating its role in promoting OP and fracture risk.^103^ Mechanistically, TMAO promotes osteoclast differentiation and bone loss by activating the ROS-dependent NF-κB signaling pathway, enhancing RANKL- and M-CSF-induced osteoclast differentiation and inflammation in RAW 264.7 cells.^104^ ^,^ ^105^ Notably, the antioxidant *N*-acetylcysteine (NAC) inhibits this pathway, suppressing TMAO-induced osteoclast formation and the expression of key genes (c-Fos/NFATc1), suggesting a therapeutic strategy.^104^ Gut microbiota dysbiosis significantly influences OP, with *Bifidobacteriaceae* impacting bone health through circulating metabolites such as phosphatidylcholine (which affects the osteogenic capacity of BM mesenchymal stem cells – BMSCs) and other choline metabolites.^106^ Supporting the therapeutic targeting of this axis, gold nanospheres (GNS) prevent OVX-induced OP in a gut microbiota-dependent manner by restoring microbial diversity, reducing TMAO abundance, and suppressing the release of pro-osteoclastogenic/pro-inflammatory cytokines TNF-*α*, IL-6, and G-CSF.^107^

#### 3.2.4. Polyamines

Polyamines are a class of low-molecular-weight aliphatic polycationic compounds (containing ≥2 amino groups), primarily including putrescine, spermidine, and spermine.^108^ They are positively charged at physiological pH and participate in key biological processes, such as cell proliferation, differentiation, and apoptosis. The gut microbiota, such as lactobacilli and bifidobacteria, synthesizes polyamines through enzymatic reactions such as arginine decarboxylase; other bacterial groups such as *E. coli*, also contribute to polyamine metabolism, albeit through different mechanisms. Beyond endogenous synthesis and dietary intake, polyamines can also be synthesized by the gut microbiota and released into the host.^109^ ^,^ ^110^ Exhibiting rapid plasma turnover and the ability to quickly reach target tissues, the fecal microbiota may correlate with fecal polyamine concentrations, and certain bacterial species within the *Clostridium* cluster XIVa may play a significant role in regulating human intestinal polyamine levels.^111^ Polyamines directly stimulate osteoblast proliferation and differentiation. Exogenous polyamines safely promote the osteogenic differentiation of human BM mesenchymal stem cells (hBMSCs) without inducing cell death or apoptosis. They achieve this by significantly enhancing alkaline phosphatase (ALP) activity and bidirectionally regulating gene expression – upregulating osteogenic genes (RUNX2, ALP, osteopontin, osteocalcin) while downregulating adipogenic genes, thereby inhibiting fat accumulation, promoting extracellular matrix mineralization, and ultimately accelerating bone formation.^112^ Polyamines inhibit osteoclast activity and prevent bone loss. They block the RANKL-induced differentiation of osteoclast precursors in a concentration-dependent manner by suppressing the NF-κB/MAPK signaling pathways, reducing the formation of multinucleated TRAP-positive cells without affecting cell survival. Polyamines directly diminish the bone-resorbing function of mature OC by inhibiting the secretion of acidic substances and hydrolytic enzymes, such as matrix metalloproteinases, thereby weakening the bone matrix degradation capacity.^113–116^ *In vivo* studies confirmed that spermidine or spermine administration significantly reduces osteoclast numbers on bone surfaces in OVX mice, prevents bone volume loss, and does not interfere with osteoblast function.^113^ These findings indicate that supplementation with natural polyamines holds preventive and therapeutic value for OP. Polyamines regulate bone-related cytokines to maintain bone metabolic homeostasis, promoting the secretion of pro-osteogenic factors such as TGF-*β* and IGF-1, which enhance osteoblast proliferation/differentiation and inhibit osteoclast activity; they also suppress the expression of pro-inflammatory cytokines such as TNF-*α* and IL-6, blocking inflammation-induced osteoclast activation and bone loss.^117^ ^,^ ^118^ Furthermore, polyamine metabolism, particularly the arginine pathway, plays a central role in regulating systemic and mucosal adaptive immunity, indirectly maintaining bone microenvironment stability by influencing the immunometabolic functions of macrophages and T cells.^119^ Probiotics modulate bone metabolism by enhancing intestinal polyamine biosynthesis – promoting osteoblast differentiation and bone formation, inhibiting osteoclast activation and bone resorption, and coordinating the balance between pro-osteogenic and anti-inflammatory factors, thereby improving OP.

#### 3.2.5. Amino acid

The gut microbiota metabolizes ingested proteins and amino acids. Firstly, it breaks down dietary proteins and peptides into amino acids for bacterial growth and proliferation. It also metabolizes tryptophan into indole derivatives, such as IAA and IPA, which act as signaling molecules regulating gut barrier function, immunity, and metabolism.^120^ Secondly, it synthesizes small amounts of essential amino acids for the host; For instance, *Escherichia coli* produces lysine, which helps maintain human amino acid balance during dietary deficiencies.^121^ The dietary amino acid composition directly influences the gut microbiota. High-protein diets, by enriching amino acid availability, promote the growth of bacteria such as Bacteroidetes and Firmicutes, thereby altering the microbial community structure.^122^ Imbalances in specific amino acids (e.g., chronic methionine deficiency) can cause dysbiosis, impair the intestinal barrier and immune function, and indirectly disrupt bone metabolism.^123^ Tryptophan-derived signaling molecules further regulate osteoblast‒osteoclast communication, coordinating the balance between bone formation and resorption to maintain bone homeostasis.^124^ Depletion of gut microorganisms significantly reduces the mechanical adaptability of bone. The gut microbiota differs between mice with varying responses to exercise, with the abundance of Lachnospiraceae directly influencing the bone's mechanical responsiveness. Microbial metabolites, specifically L-citrulline and its conversion to L-arginine, are critical for bone's mechanical adaptation. Supplementation with these substances enhances the bone's mechanical response in normal, aged, and OVX mice. Mechanistically, L-arginine acts by activating a nitric oxide-Ca ion positive feedback loop in osteocytes. This research reveals the microbiota‒metabolite axis as a potential novel strategy against OP.^125^

##### 3.2.5.1. Promotion of osteoblast activity

Arginine and glutamine together promote osteoblast activity and bone formation. Arginine promotes collagen synthesis by OB and enhances bone formation by activating the mTOR pathway.^126^ Glutamine, via transglutaminase 2 (TGM2), improves mitochondrial dynamics and ATP production, driving osteogenic differentiation and improving OP.^127^ Skeletal stem cells (SSCs) rely on glutaminase (GLS) and *α*-ketoglutarate (*α*-KG) to regulate the osteogenic‒adipogenic differentiation balance; activating GLS can increase bone mass during aging.^127^ M2 macrophage-derived extracellular vesicles (M2-EVs) enhance glutamine metabolism in osteoclast precursors (OCPs), reprogramming them into M2-like cells via *α*-KG/Jmjd3-mediated epigenetic mechanisms to suppress bone resorption.^128^ Targeted inhibition of GOLM1 (which promotes glutamine metabolism via mTOR activation) or its downstream pathways can reverse the inhibition of BM stromal cell (BMSC) osteogenic differentiation and improve OP.^129^ However, the GLS inhibitor CB-839 exhibits a bidirectional effect: it counteracts bone loss in OVX models but exacerbates bone loss in aging models. This dual effect stems from the differential regulation of downstream metabolic pathways within glutaminolysis.^130^

##### 3.2.5.2. Inhibition of osteoclast formation and activity

The gut microbiota can inhibit osteoclast activity and reduce bone resorption through amino acid metabolic pathways. Leucine and its metabolite *α*-ketoisocaproate (*α*-KIC) can inhibit the differentiation and maturation of osteoclast precursor cells and reduce the bone resorption activity of OC by regulating the NF-κB signaling pathway.^114^ Simultaneously, polyamines, as downstream products of amino acid metabolism, can also interfere with osteoclast function. Studies have found that polyamines can inhibit the expression of bone resorption-related genes in OC, reduce the secretion of acidic substances and proteases by OC, thereby maintaining bone metabolic balance. CARM1 drives osteogenic differentiation and reduces bone loss by dual mechanisms: it promotes glycolysis and inhibits oxidative phosphorylation. It achieves this by methylating the PPP1CA-R23 site to regulate AKT/AMPK dephosphorylation and acting as a transcriptional co-activator to inhibit PDH activity.^131^ Glu is an essential substrate for osteoclast differentiation and function. V9302, an inhibitor of its transporter ASCT2, suppresses osteoclast differentiation and bone resorption by depriving cells of Glu, and improves bone loss in OVX mice. Mechanistically, Glu mediates IL-17's promotion of osteoclast energy metabolism. The bone-loss exacerbating effect of IL-17 depends on Glu and its metabolite *α*-ketoglutarate (*α*-KG), revealing that the IL-17-Glu-*α*-KG metabolic axis is a therapeutic target.^132^ ^,^ ^133^ Glutaminolysis provides key amino acids and nucleotides for OC. Both genetic and pharmacological evidence (using the inhibitor CB-839) confirms that it regulates OC and bone resorption both in vitro and in vivo. Inhibiting glutaminolysis can prevent and treat OVX-induced bone loss.^132^ Research has found that Glu is an essential substrate for osteoclast differentiation and function. The ASCT2 transporter inhibitor V9302 suppresses osteoclast activity and ameliorates bone loss in OVX models by depriving cells of Glu. Mechanistically, Glu mediates IL-17's promotion of osteoclast energy metabolism, and IL-17's exacerbation of bone loss depends on Glu and its metabolite *α*-ketoglutarate (*α*-KG). This reveals the “IL-17-Glu-*α*-KG metabolic axis” as a therapeutic target.^133^ Simultaneously, glutaminolysis regulates osteoclast metabolic reprogramming, differentiation, and bone resorption by supplying key amino acids and nucleotides (supported by in vitro and in vivo evidence). Inhibiting this pathway (e.g., using the glutaminase inhibitor CB-839) effectively prevents and treats OVX-induced bone loss, providing genetic and pharmacological validation for targeting glutamine metabolism.^132^

##### 3.2.5.3. Impact on the bone microenvironment

Oxidative stress exacerbates OP through excessive ROS production, while amino acid metabolism and its derivatives can maintain the redox balance in the bone microenvironment. Cysteine, as a precursor of glutathione (GSH), is regulated by the gut microbiota and elevates intracellular GSH levels, thereby scavenging ROS. PINK1 deficiency drives excessive osteoclast activation via the mitochondrial ROS/NFATc1 signaling pathway, manifesting as reduced bone mass and increased TRAP-positive cells. Spermidine (SPD) and *N*-acetylcysteine (NAC) can restore mitophagy and suppress oxidative stress, respectively.^134^ Concurrently, OP disrupts intestinal tryptophan metabolism and reduces microbiota-derived melatonin (MLT). Exogenous MLT ameliorates bone loss via the “gut‒bone axis” by restoring the microbiota (increasing the abundance of probiotics *Allobaculum* and *Parasutterella*), promoting SCFAs production, inhibiting TMAO metabolism, balancing M1/M2 macrophage polarization, and repairing the intestinal barrier.^29^

##### 3.2.5.4. Promotion of angiogenesis and mitophagy

L-arginine alleviates bone loss and synergistically promotes osteogenesis and angiogenesis. Its mechanisms include enhancing vascular endothelial cell activity, inhibiting adipogenesis, and inducing mitophagy by regulating the expression of PINK1/Parkin and Bnip3 in osteoblasts and endothelial cell lines, thereby protecting cells from ROS damage.^135^ Meanwhile, tryptophan metabolites (IAA/IPA) regulate bone homeostasis via the AhR-gut-bone axis. Supplementation with IAA and IPA repairs the intestinal barrier in OVX mice in an AhR-dependent manner by activating the Wnt/β-catenin pathway. It also promotes IL-10 secretion by M2 macrophages, extending from the gut lamina propria to the bone marrow, thereby promoting osteogenesis while suppressing OC both *in vivo* and *in vitro*. This effect was abolished in intestinal AhR-knockout OVX mice, confirming that microbial tryptophan metabolites represent a promising therapeutic target for OP through the AhR-mediated “gut‒bone axis”.^136^

### 3.3. Intestinal barrier

The integrity of the intestinal barrier is essential for bone health. Its four-layer structure, comprising mechanical, chemical, immune, and biological barriers, synergistically defends against pathogen invasion. During sex hormone deficiency, particularly in OVX models, downregulated expression of the tight junction proteins ZO-1, Occludin, and Claudin-1 increases intestinal permeability. This promotes bacterial metabolite translocation, activates Th17 cells, and upregulates osteoclastogenic factors, including TNF-*α*, IL-17, and RANKL in the bone marrow, driving bone loss.^137^ Antibiotic-induced dysbiosis featuring an elevated Firmicutes-to-Bacteroidetes ratio similarly exacerbates permeability and trabecular bone loss. Conversely, probiotics such as *Lactobacillus reuteri* and *Bifidobacterium infantis*, or the mucus supplement MDY-1001, effectively reverse barrier damage and restore bone metabolic balance by repairing tight junctions, reducing inflammatory factor IFN-*γ*, and modulating the microbial composition.^138^ ^,^ ^139^ In conclusion, targeting the gut barrier through probiotic interventions can block the microbiota-inflammation-bone loss axis, offering novel strategies for OP prevention and treatment.

The gut microbiota maintains intestinal barrier function through metabolites and immune regulation. Healthy microbiota-derived SCFAs enhance tight junction protein expression by activating GPR41/43 receptors and HDAC pathways, supplying energy to intestinal epithelial cells while inhibiting pro-inflammatory cytokine secretion. Dysbiosis, conversely, disrupts barrier integrity, increasing permeability. Simultaneously, the microbiota regulates immune balance within the GALT, inducing Treg cell differentiation and promoting anti-inflammatory cytokine IL-10 secretion, thereby reducing the levels of pro-inflammatory factors such as TNF-*α* and IL-6. Probiotics, including *Bifidobacterium* species and *Lactobacillus rhamnosus* CNCM I-3690, can restore barrier damage by upregulating the tight junction proteins ZO-1, Occludin, and Claudin-3, clearing ROS, and rebalancing the gut microbiota. Their efficacy is comparable to that of the beneficial commensal bacterium *Faecalibacterium prausnitzii* as demonstrated in previous studies.^140–143^ The intestinal microbiota regulates the mechanism by which the intestinal barrier plays a role in OP in the following ways.

#### 3.3.1. Relief of inflammation and inhibition of OC

Intestinal barrier dysfunction can lead to the entry of enterogenous inflammatory factors into the bloodstream, induce systemic inflammation, activate the differentiation of OC, and promote bone resorption. By strengthening the intestinal barrier, the gut microbiota attenuates systemic inflammation through the suppression of pro-inflammatory cytokines, thereby inhibiting osteoclastogenesis and bone resorption while concurrently enhancing osteoblast differentiation and bone formation. Studies have shown that in OVX mice, the abundance of Order_Burkholderiales sharply decreases, *Ruminococcus* significantly increases, the level of intestinal SIgA drops, the expression of inflammatory factors rises, the barrier is damaged, and the content of LPS in the colon and serum increases significantly.^144^ The gut microbiota regulates LPS levels through the bone‒gut axis to improve the immune microenvironment, thereby influencing the pathological process of OP. This mechanism involves complex interactions among microbial metabolites, intestinal barrier integrity, innate immune signaling pathways, and bone metabolic balance. The core mechanism begins with LPS produced by Gram-negative bacteria (such as *Bacteroides*) translocation through the damaged intestinal barrier into the circulatory system, activating Toll-like receptor 4 (TLR4) on the surface of monocytes/macrophages, triggering the NF-κB signaling pathway to upregulate the expression of pro-inflammatory cytokines (TNF-*α*, IL-6, and IL-1β). These inflammatory factors reach the bone microenvironment through the blood circulation. On the one hand, they directly inhibit osteoblast differentiation by suppressing the phosphorylation of the key protein LRP5/6 in the Wnt/β-catenin pathway. On the other hand, they promote osteoclast generation by up-regulating the RANKL/OPG ratio and enhancing RANK and M-CSF signals. Ultimately, this leads to an imbalance between bone resorption and formation.^145–147^

Specifically, *Bifidobacterium longum* CCFM1076 can reduce serum LPS levels by competitively inhibiting the colonization of LPS-producing flora.^148^ Preclinical studies have shown that a 6-week intervention with the probiotic VSL#3 (containing 8 strains of lactic acid bacteria and *Bifidobacterium*) can increase the femoral BMD in OVX mice. The mechanism is related to the reduction of serum LPS, the inhibition of the decline in TRAP activity of OC, and the upregulation of the expression of the osteogenic marker Runx2.^149^ It is worth noting that *Akkermansia muciniphila* reduces LPS translocation by increasing the thickness of the mucus layer and upregulating the expression of Muc2 protein. Meanwhile, its membrane protein Amuc_1100 can directly activate TLR2 signaling and promote the secretion of GLP-2 by intestinal L cells. The latter stimulates osteoblast proliferation through the *β*-catenin pathway.^150^ ^,^ ^151^ At the molecular level, LPS-TLR4 signaling can induce OB to express SOST/sclerostin and inhibit the nuclear translocation of the key Wnt pathway protein *β*-catenin. Meanwhile, prebiotic fructooligosaccharide intervention can reduce the level of sclerostin in serum by increasing the prevalence of *Bifidobacterium* and restoring the rate of bone formation.^152^ These findings suggest that targeting the gut microbiota-LPS-immune axis may become a new strategy for OP treatment. Future research needs to further clarify the precise mechanisms by which specific strain-derived metabolites, such as indole derivatives and polyamines, regulate bone immunity.

#### 3.3.2. Promotion of metabolite absorption

Metabolites of the gut microbiota, such as SCFAs, promote Ca absorption by lowering the intestinal pH (increasing Ca ion solubility) and upregulating the Ca transporter protein TRPV6. Improved intestinal barrier function further enhances nutrient absorption efficiency, providing sufficient Ca ions for bone mineralization. Increased concentrations of the probiotics *Lactobacillus reuteri* and *Bifidobacterium longum* in the gut may increase BMD by promoting the absorption of minerals (calcium, magnesium, and phosphates).^153^ Nodakenin treatment effectively improves the bone microstructure, elevates the serum levels of bone turnover markers, and enhances intestinal mucosal integrity. 16S rRNA sequencing analysis revealed that this treatment reduces the relative abundance of Firmicutes and Patescibacteria and the F/B ratio, while increasing the relative abundance of Bacteroidetes in OVX mice. It also elevates the relative abundance of genera such as Muribaculaceae and *Allobaculum*. Metabolomic analysis indicates that nodakenin significantly alters 113 metabolites, including calcitriol. Correlation analysis demonstrates strong links between the gut microbiota, OP phenotypes, and metabolites. Nodakenin treatment modulates the gut microbiota, repairs the intestinal barrier, and alleviates OVX-induced OP.^154^ Probiotic administration significantly prevents inflammatory alveolar bone resorption in OVX rats. By enriching butyrate-producing genera and enhancing intestinal butyrate production, probiotics improve the intestinal barrier and reduce intestinal permeability in OVX rats. Furthermore, the estrogen deprivation-induced inflammatory response is suppressed in probiotic-treated OVX rats, as reflected in reduced serum levels of inflammatory cytokines and a balanced distribution of CD^4+^ IL-17A^+^ Th17 cells and CD^4+^ CD^25+^ Foxp^3+^ Treg cells in the BM.^155^ SCFAs produced by butyrate-generating bacteria (e.g., *Faecalibacterium*) from dietary fiber not only inhibit the NF-κB pathway via GPR43 activation (reducing IL-6 by 62%) but also promote regulatory T cell (Treg) differentiation and IL-10 secretion, thereby suppressing excessive bone resorption.^156^ Intestinal mucosal barrier dysfunction may lead to elevated serum LPS levels, which in turn increase intestinal permeability and ultimately contribute to bone loss.^157^

#### 3.3.3. Regulation of bone metabolism hormones

The gut microbiota influences bone metabolism and hormone levels through the enteroendocrine system. Incretins such as GLP-1 and PYY indirectly promote bone formation by modulating insulin secretion. Additionally, microbiota-mediated regulation of bile acid metabolism affects vitamin D activation and Ca metabolism, thereby improving bone health. Studies have demonstrated that GLP-2 ameliorates age-related bone loss, enhances intestinal tight junction integrity, and boosts intestinal antioxidant enzyme activity in SAMP6 mice. Improving gut barrier dysfunction may facilitate bone formation, providing a rationale for targeting the gut‒bone axis in senile OP therapy.^158^

#### 3.3.4. Alleviation of oxidative stress

Intestinal barrier damage may increase endogenous ROS production in the gut,^159^ exacerbating skeletal oxidative damage.^160^ A healthy gut microbiota mitigates bone metabolic disorders by metabolizing antioxidants and reducing ROS generation.^161^

### 3.4. Endocrine system

The gut microbiota exerts a positive influence on OP through the endocrine system. It participates in the enterohepatic circulation of estrogen: specific bacterial *β*-glucuronidase enzymes hydrolyze conjugated estrogen into its free form for reabsorption, maintaining systemic estrogen levels to support BMD. Microbial metabolites, particularly SCFAs such as propionate and butyrate, activate G protein-coupled receptors to modulate gut hormone secretion. This indirectly promotes estrogen synthesis and signaling while inhibiting aromatase activity, thereby reducing androgen-to-estrogen conversion and optimizing bone metabolism. Concurrently, the gut microbiota regulates the metabolism of PTH and vitamin D, ensuring calcium‒phosphate homeostasis and skeletal health. Consequently, targeting the gut microbiota to modulate the endocrine system represents a crucial therapeutic strategy for preventing and treating OP.

#### 3.4.1. Estrogen

Estrogen deficiency is a universal challenge for postmenopausal women and leads to OP. Estrogen is a crucial hormone for maintaining BMD and preventing OP. It reduces bone resorption by inhibiting osteoclast activity and promotes bone formation by enhancing osteoblast function. Estrogen receptors (ERs) are highly expressed in bone cells and promote bone formation by regulating target genes such as IGF1 and TGF-*β*. During estrogen deficiency, ER dysfunction leads to increased secretion of T cell-mediated cytokines such as IL-1, IL-6, and TNF-*α*, which indirectly enhances OC differentiation and triggers OP.^162^ The gut microbiota plays a pivotal role in the enterohepatic circulation of estrogen. Endogenous and exogenous estrogens enter the enterohepatic circulation and are first metabolized in the liver (glucuronidation or sulfation), forming water-soluble conjugates that are subsequently excreted into the intestine via bile.^163^ In the intestine, bacterial *β*-glucuronidase enzymes hydrolyze these conjugated estrogens, converting them back to their free form. Free estrogen can then be reabsorbed into the bloodstream, elevating serum estrogen levels and completing the enterohepatic cycle. Gut dysbiosis reduces the activity of this enzyme, leading to decreased estrogen reabsorption, increased excretion, lower systemic estrogen levels, and an elevated risk of OP.^164^ ^,^ ^165^ Furthermore, SCFAs produced by the gut microbiota not only promote the secretion of gut hormones such as glucagon-like GLP-1,^69^ thereby influencing estrogen synthesis and signaling, but also inhibit aromatase activity in adipose tissue. This reduces the conversion of androgens to estrogen, optimizing bone metabolic balance.^166^ ^,^ ^167^ Regarding bone metabolism, estrogen exerts critical effects primarily through its receptors (ERs). The estrogen receptor complex in osteoblast progenitor cells activates the Wnt/β-catenin signaling pathway, promoting osteogenesis.^168^ Estrogen also upregulates bone morphogenetic protein (BMP) signaling, driving the differentiation of mesenchymal stem cells into pre-OB and osteoblasts. Simultaneously, estrogen receptors influence bone resorption by regulating key factors in OC: they inhibit the action of receptor activator of nuclear factor kappa-B ligand (RANKL binding to its receptor RANK activates OC) and promote the expression of OPG (OPG acts as a decoy receptor for RANKL, inhibiting its activity). This effectively suppresses osteoclast formation and bone resorption function.^169^ ^,^ ^170^

#### 3.4.2. PTH

The gut microbiota bidirectionally regulates bone metabolism by modulating PTH. Intermittent PTH exposure promotes osteoblast-mediated bone formation, whereas sustained high concentrations of PTH activate OC, leading to bone loss. In patients with primary hyperparathyroidism (PHPT), the abundance of the *Subdoligranulum* is significantly increased and positively correlates with lumbar spine BMD. Conversely, the abundance of genera such as *Ruminococcus* and *Alloprevotella* is reduced. This dysbiosis reverses following parathyroidectomy. Furthermore, the abundance of *Clostridium_sensu_stricto_1* negatively correlates with the serum Calevels and positively correlates with the femoral neck BMD.^171^ Intermittent PTH treatment remodels the gut microbiota in OVX rats. It increases the abundance of beneficial bacteria such as *Lactobacillus reuteri* and Muribaculaceae, suppresses Rikenellaceae, and promotes butyrate synthesis, ultimately enhancing BMD.^172^ Targeted modulation of probiotics and butyrate metabolism to influence PTH signaling provides novel therapeutic avenues for the prevention and treatment of OP.

##### 3.4.2.1. Immune modulation

A healthy gut microbiota helps maintain immune balance and reduces chronic inflammation. Chronic inflammation can disrupt PTH secretion, subsequently affecting bone metabolism. For instance, inflammatory cytokines IL-6 and TNF-*α* can stimulate PTH secretion and exacerbate bone resorption. Notably, PTH's stimulation of bone formation and increase in bone mass depend on the gut microbiota and its metabolites. A key metabolite, butyrate, is essential for CD^4+^ T cell differentiation, regulatory Treg cell expansion, and the secretion of the osteogenic factor Wnt10b by CD^8+^ T cells.^173^ When microbiota depletion leads to reduced butyrate, PTH's bone-anabolic effects are lost; however, supplementing physiological levels of butyrate restores this effect.^174^ The mechanism lies in PTH requiring butyrate (acting through the dendritic cell GPR43 and T cell non-GPR43 pathways) to increase BM Treg numbers. Tregs then stimulate BMCD^8+^ T cells to produce Wnt10b, activating the Wnt pathway to promote bone formation. Meanwhile, specific gut bacteria, such as segmented filamentous bacteria (SFB), which induce Th17 cells, mediate the detrimental bone loss effects of PTH, a common complication of hyperparathyroidism.^175^ Studies show that PTH induces significant bone loss only in the presence of SFB. When SFB is present, PTH increases intestinal TNF-producing T cells and Th17 cells. These cells migrate from the gut to the BM via activation of S1P receptor-1. CXCR3 mediates the homing of TNF-producing T cells to the bone marrow, where they upregulate the chemokine CCL20. This further recruits more Th17 cells into the bone marrow. Together, these cells exacerbate bone loss. Therefore, targeting the modulation of gut microbiota composition or intervening in the migration of T cells to the BM holds promise as a novel strategy for treating hyperparathyroidism-associated bone loss.

##### 3.4.2.2. Promoting vitamin D absorption

The gut microbiota influences vitamin D metabolism by modulating its metabolic pathways, thereby affecting the generation of its active form, 1,25-dihydroxyvitamin D₃ (1,25(OH)₂D₃). 1,25(OH)₂D₃ promotes intestinal Ca absorption and suppresses PTH secretion. PTH, in turn, also promotes the conversion of 25-hydroxyvitamin D (25(OH)D) to 1,25(OH)₂D₃ in the kidneys, forming a feedback loop. A healthy gut microbiota helps maintain the normal functioning of this cycle, ensuring the balance of Ca and phosphorus metabolism.^176^

##### 3.4.2.3. Increasing Ca absorption

The gut microbiota influences Ca absorption. The gut microbiota ferment dietary fiber to produce SCFAs. These SCFAs not only provide energy for intestinal epithelial cells but also enhance intestinal barrier function, reduce inflammatory responses, and promote Ca absorption. SCFAs can increase the expression of calcium-binding proteins (such as Calbindin-D9k) in intestinal epithelial cells, further improving Ca absorption efficiency. High levels of Ca absorption help maintain blood Ca homeostasis and reduce excessive PTH secretion. Dietary Ca is transported across the mucosal epithelium via both saturable transcellular and non-saturable paracellular pathways, both of which are regulated by 1,25-dihydroxyvitamin D and PTH.^177^

##### 3.4.2.4. Regulation of the hypothalamic-pituitary-thyroid (HPT) axis via the gut–brain axis

The gut microbiota regulates the HPT axis through the gut‒brain axis. Its metabolites can modulate PTH secretion and activity via vagus nerve signaling. A randomized double-blind controlled trial (*n* = 50) in osteopenic patients aged 50–72 y demonstrated that supplementation with a multi-species probiotic for 6 months resulted in significantly reduced levels of bone metabolism markers (bone-specific alkaline phosphatase, BALP, and C-terminal telopeptide of type I collagen, CTX) in the probiotic group compared to the placebo group. Concurrently, serum PTH and the inflammatory cytokine TNF-*α* levels decreased.^178^ This indicates that probiotics may improve bone metabolism by modulating PTH. Future strategies could include dietary interventions (such as increasing dietary fiber intake) to promote the proliferation of beneficial bacteria and enhance SCFAs production, thereby optimizing Ca absorption and PTH balance.

**Figure 4.**
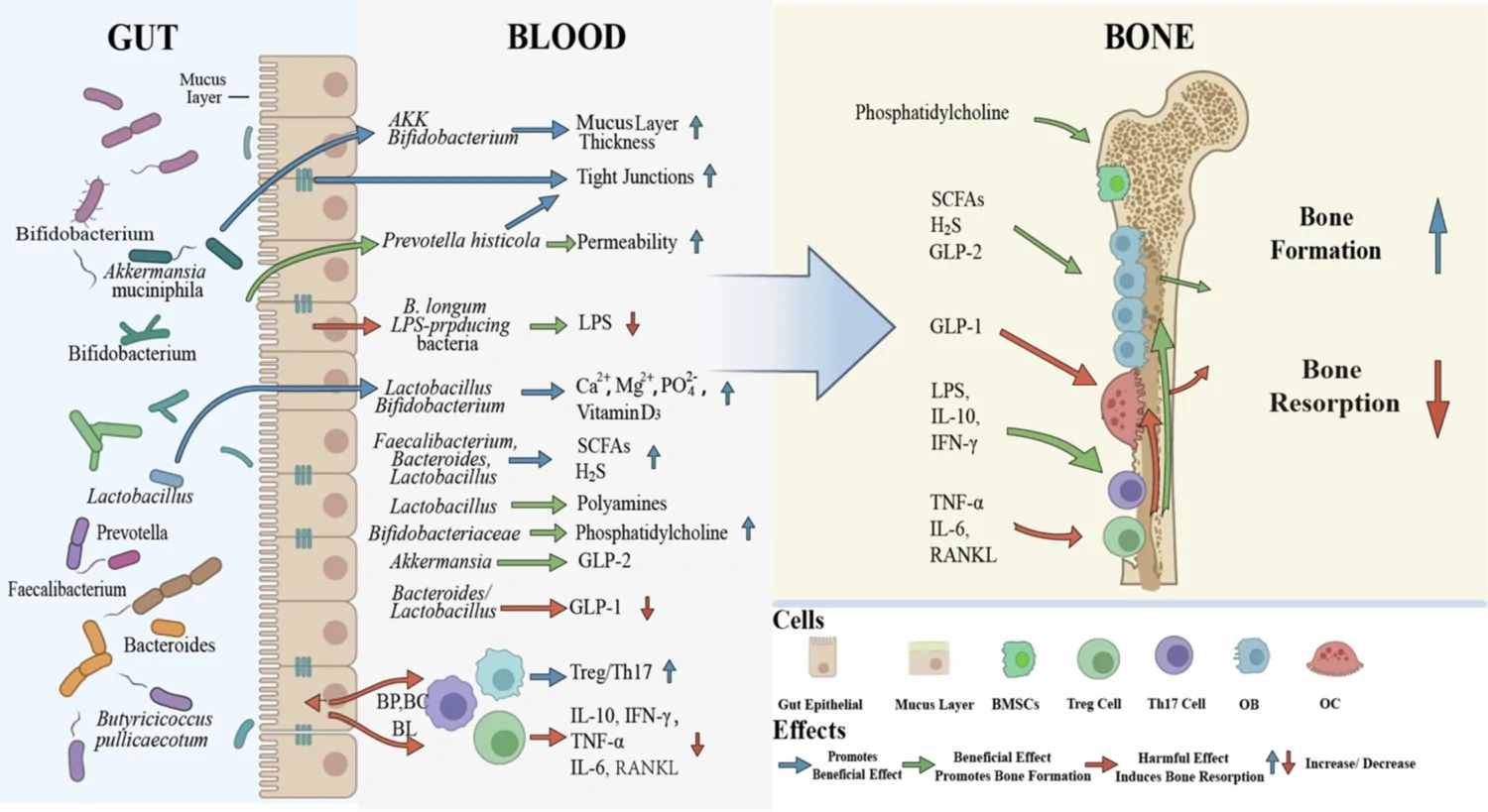
The gut–bone axis mechanism: microbiota-mediated pathways modulating intestinal biomechanics and bone remodeling.

Overall, accumulating evidence indicates that the gut microbiota regulates bone metabolism not only through immune and metabolic pathways, but also by modulating intestinal biomechanics. Specific bacterial groups can alter intestinal barrier integrity, the mucus layer structure, epithelial permeability, and gut motility, thereby reshaping the mechanical and biochemical microenvironment of the intestine. These biomechanical changes influence nutrient absorption, immune activation, and microbial metabolite signaling, which collectively affect osteoclast–osteoblast coupling and bone remodeling. To integrate these complex interactions, a schematic diagram is presented to summarize how distinct bacterial taxa impact intestinal biomechanics and subsequently regulate bone metabolism through the gut–bone axis (Figure 4).

Specific gut bacterial taxa regulate bone metabolism by reshaping the mechanical and biochemical microenvironment of the intestine. Alterations in intestinal barrier integrity, mucus layer structure, epithelial permeability, and gut motility directly influence nutrient absorption (e.g., calcium), local and systemic immune activation, and the signaling of microbial metabolites (such as SCFAs and bile acids). These integrated biomechanical and biochemical changes subsequently coordinate the coupling between osteoclasts and osteoblasts, thereby modulating overall bone remodeling.

## 4. Research progress of probiotics in OP treatment

### 4.1. Animal experimental studies

Recent animal studies have demonstrated that probiotics serve as a beneficial dietary supplement for humans, not only enhancing BMD in healthy individuals but also exerting significant effects on the prevention and treatment of postmenopausal OP.^179^ Commonly utilized probiotic strains in this field include *Bifidobacterium, Lactobacillus,* and *Lactobacillus reuteri.* Current animal experiments primarily involve bilateral ovariectomy in standard rats to establish OP models, followed by the administration of the corresponding probiotics. The efficacy of probiotics in preventing and treating osteoporosis is evaluated through measurements of bone metabolism indices, the gut microbiota composition, immune cell profiles, inflammation-related factors, calcium absorption indices, and hormone levels. Research has confirmed that probiotic treatment can effectively improve bone metabolism indicators, enhance bone microstructure by increasing BMD and bone mass, promote bone formation via the regulation of bone metabolism markers, and inhibit bone resorption^180^ (Table 2). For instance, *Bifidobacterium* can modulate osteoclast formation by inhibiting the TNF-α/NF-κB inflammatory pathway through its secreted products, including short-chain fatty acids (SCFAs) and extracellular polysaccharides (EPS), thereby preventing osteoclast-induced bone resorption and OP.^181^ Recent studies have demonstrated that osteoporotic animals frequently exhibit intestinal microbiota imbalance. Probiotics can modulate the microbial community, increasing the abundance of beneficial bacteria such as Lactobacillaceae, Ruminococcaceae, Bifidobacteriales, and Rikenellaceae, while decreasing the presence of potentially harmful taxa, including certain Firmicutes, Desulfobacterota, Lachnospiraceae, Oscillospiraceae, and Desulfovibrionaceae, thereby restoring microbial equilibrium.^35^ ^,^ ^182^ Among these, *Lactobacillus,* a key probiotic in the gut microbiome, plays a crucial role in maintaining intestinal barrier integrity and modulating immune responses.^183^ Additionally, probiotics can reduce intestinal permeability and enhance barrier function by promoting the expression of tight junction proteins (ZO-1, Occludin, claudin, and GLP-2), thereby preventing inflammatory factors from entering the bloodstream and mitigating systemic inflammation.^21^ Research has also shown that intestinal *Lactobacillus* can activate the Wnt signaling pathway via hydrogen sulfide production, promoting osteoblast formation and inhibiting osteoblast apoptosis.^184^ Furthermore, probiotics exert positive effects on immune regulation by inhibiting inflammatory responses, modulating immune cell activity, and suppressing the release of pro-inflammatory cytokines such as TNF-*α* and IL-6, thus reducing the detrimental impact of inflammation on bone metabolism.^185^ Probiotics have been shown to induce the differentiation of regulatory T cells, enhance immune tolerance, and mitigate the adverse effects of autoimmune responses on bone tissue. For instance, *Bacillus clausii* has been demonstrated to modulate the Treg‒Th17 balance in mice by inhibiting the differentiation of Th17 cells from osteoclast precursors and promoting the development of anti-osteoclastogenic Treg cells in a postmenopausal osteoporosis model, thereby confirming its osteoprotective potential.^39^ Additionally, research indicates that probiotics can produce SCFAs, lower the intestinal pH, and enhance calcium absorption.^186^ Furthermore, probiotics may indirectly influence bone metabolism through the regulation of estrogen and parathyroid hormone (PTH) levels.^187^

### 4.2. Clinical trial research progress

While several animal studies have demonstrated the potential of probiotics in osteoporosis treatment, clinical trials remain in their infancy, with a limited number of studies and variable outcomes. Most current clinical research employs *Lactobacillus* and *Bifidobacterium*, either as single strains or in combination. Intervention doses and study durations varied across trials. The majority of participants were postmenopausal women or elderly patients diagnosed with OP. The key outcome measures included BMD, bone metabolic markers, the gut microbiota composition, and inflammatory factors (Table 3). Some studies have indicated that probiotic supplementation can significantly increase BMD in postmenopausal women compared with placebo. For instance, a clinical trial involving *Lactobacillus reuteri* ATCCPTA 6475 (*L. reuteri* 6475) in postmenopausal women with osteopenia demonstrated a reduction in total tibial volumetric BMD (vBMD) loss compared with that of placebo (mean difference: 1.02%, 95% CI: 0.02–2.03; *P* = 0.043).^188^ However, another trial using the same genus, but different dosages, found no significant differences in tibial vBMD, hip and spine vBMD, tibial cortical area, or BMD compared with placebo. Additionally, some studies have reported that probiotics can reduce the serum levels of bone resorption markers and increase the levels of bone formation markers. A novel isoflavone aglycone and probiotic-rich red clover extract (RCE) administered to postmenopausal women with osteopenia resulted in a significant reduction in BMD loss associated with estrogen deficiency (*P* = 0.043) and notable improvements in bone turnover markers (*P* = 0.045).^189^ A study demonstrated that *Bifidobacterium animalis* significantly enhances bone metabolism, increases the serum vitamin D3 concentration, and decreases the serum PTH and procalcitonin levels in postmenopausal women with osteopenia (*P*＜0.05).^190^ Additionally, *Bifidobacterium* lactis Probio-M8 influences gut microbial interactions, particularly those involving short-chain fatty acid-producing bacteria. The expression of genes encoding various carbohydrate metabolic pathways, including ABC transporters, phosphotransferase systems, and pathways for fructose and mannose metabolism, as well as choline phosphocytidyl transferase, was notably elevated.^190^ Furthermore, research has indicated that probiotics can reduce the serum levels of inflammatory markers such as C-reactive protein, IL-6, TNF-*α*, and IL-1β. In a study by Sadegh Jafarnejad et al., the intervention group exhibited statistically significant reductions in serum PTH and TNF-*α* compared with the placebo group (*P*＜0.05).^178^ Although probiotics have demonstrated promising efficacy in animal studies, the outcomes of clinical trials remain inconsistent, with some investigations failing to show significant improvements in OP. Currently, the scale of clinical studies in this field is limited, and the optimal dosage and treatment duration for probiotics have yet to be conclusively established. Additionally, the primary mechanisms and effects of probiotics on the human body require further elucidation. Future clinical research will prioritize a deeper exploration of their mechanisms of action, particularly focusing on immune modulation and intestinal barrier function. Moreover, the development and screening of novel probiotic strains hold considerable promise for advancing the prevention and treatment of OP.

**Table 2.** Literature on the results and mechanisms of probiotics in the treatment of osteoporosis in animal models.

Probiotic	Animal model	Treatment dose	Human equivalent dose (HED)	Treatment duration	Skeletal outcomes	Immune effects	Gut barrier effects	Microbiota modulation	Proposed mechanism/conclusion	Ref.
*Lactobacillus rhamnosus* GG (ATCC 7469)	OVX-induced OP (female Sprague-Dawley rats)	1 × 10^9^ CFU/day by gavage (10^9^ CFU/mL; 1 mL/day)	1.6 × 10^8^ CFU/mL	6 weeks	BMD, BV/TV, BS/TV, Tb.N, Tb.Th ↑; Tb.Sp ↓; PINP ↑; CTX-I ↓	IL-17 ↓; IL-10, TGF-*β* ↑; Th17/Treg ratio ↓	ZO-1, occludin, claudin-1, GLP-2R ↑; serum LPS ↓	Firmicutes, Desulfobacterota, Lachnospiraceae, Oscillospiraceae, *Desulfovibrionaceae* ↓; Lactobacillaceae, Ruminococcaceae ↑	Th17/Treg↓ Gut microbiota structure change	[40]
*Bacillus clausii* (BC)	OVX-induced OP (female BALB/c mice)	1 × 10^9^ CFU/mL; 200 μL/day, oral	1.6 × 10^8^ CFU/mL	6 weeks	Osteoclast formation ↓; BMD, BV/TV, Tb.Th, Tb.N, Conn.D ↑; Tb.Sp, Tb.Pf ↓	Treg ↑; Th17 ↓; IL-6, IL-17, IFN-*γ*, TNF-*α* ↓; IL-10 ↑	/	/	Th17/Treg↓	[43]
Heat-killed *Limosilactobacillus reuteri* ATCC PTA 6475	OVX-induced OP (female BALB/c mice)	1.3 × 10^9^ CFU/day by gavage	2.1 × 10^8^ CFU/mL	4 weeks (0.3 mL/day)	BV/TV ↑; Tb.Th ↑	IL-6, TNF-α↓	Occludin ↓	/	Gut Occludin ↑ Intestinal IL-6 and TNF-*α* gene expression↑	[185]
Heat-killed *Lacticaseibacillus paracasei* GMNL-653	OVX-induced OP (female ICR mice)	1 × 10^10^ cells/mL, heat-killed; once daily	1.6 × 10^8^ CFU/mL	4 weeks	BV/TV, BMD ↑; TRAP-5 ↓; BMP-2: upward trend	TGF-*β*, IL-10 ↑; RANKL mRNA, serum IL-17A ↓	Serum LPS ↓; intestinal barrier integrity maintained	Tenericutes, Mollicutes ↓; Bifidobacteriales, Bifidobacteriaceae, Rikenellaceae ↑	RANKL and IL-17A ↓ Intestinal microbiota were alleviated.	[182]
*Bifidobacterium longum* UBBL-64 (M1395)	OVX-induced OP (female C57BL/6J mice)	1 × 10^9^ CFU/day by oral gavage (400 μL/day)	1.6 × 10^8^ CFU/mL	6 weeks	Bone resorption pits ↓; BV/TV, Tb.Th, BMD ↑; bone microarchitecture and strength improved	CD19⁺CD1dʰⁱCD5⁺IL-10⁺ Bregs ↑; CD4⁺Foxp3⁺IL-10⁺ Tregs ↑; CD4⁺RORgt⁺IL-17⁺ Th17 cells ↓; IFN-*γ*, IL-10 ↑; IL-6, IL-17, TNF-*α* ↓	/	/	Breg-Treg-Th17	[47]
*Prevotella histicola* (Ph)	OVX-induced OP (female C57BL/6 mice)	200 μL/day by oral gavage	/	8 weeks	BMD, BS/TV, Tb.N, SMI ↑; OPN, OPG, RUNX2 ↑	IL-1β, TNF-*α*, RANKL ↓	ZO-1, occludin ↑; intestinal mucosal barrier improved	Prevotellaceae ↑; gut microbial composition, abundance, and diversity improved	Remodeling GM Inhibiting the release of pro-inflammatory factors	[27]

**Table 3.** Literature on the results and mechanisms of probiotics in the treatment of osteoporosis in population-based studies.

Intervention	Population	Study size, n	Mean age, years	Treatment dose	Treatment duration	Main outcomes	Ref.
*Lactobacillus reuteri* ATCC PTA 6475	Women aged 75–80 y with low BMD	90	76.4	1 × 10^10^ CFU/day, orally	12 months	vBMD loss ↓ (~1.0%) (ITT: *P* = 0.043)	[188]
Red clover extract (RCE) rich in isoflavone aglycones + probiotics	Postmenopausal women with osteopenia	78	60.84	60 mg isoflavone aglycones/day + probiotics	12 months	The BMD (BMD) loss associated with estrogen deficiency↓ (*P* = 0.043) Bone turnover markers demonstrated notable ↑ (*P* = 0.045)	[189]
*Bifidobacterium animalis* subsp. *lactis* Probio-M8	Patients with postmenopausal osteoporosis	40	62.77	1.5 × 10^10^ CFU/day + calcium 600 mg/day + calcitriol 0.25 μg/day	3 months	Bone metabolism↑ (*P* < 0.05) Serum vitamin D3 concentrations↑ (*P* < 0.05) Serum PTH↓ (*P* < 0.05) Procalcitonin levels were reduced.	[190]
Enriched dairy product containing *Lactobacillus plantarum* 3547	Healthy menopausal women at risk of osteoporosis or with untreated osteopenia	78	56	1 serving/day of enriched reconstituted dairy product	24 weeks (6 months)	BMD↑ (*P* < 0.05) P1NP↑ (*P* < 0.05) CTx↓ (*P* < 0.05)	[191]
Soymilk–honey fermented with *Lactobacillus plantarum* 1 R 1.3.2	Postmenopausal women	54	57.61	100 mL/day	90 d (3 months)	Serum bone calcitonin levels ↓ (*P* < 0.05)	[192]

Abbreviations: ↑, increase; ↓, decrease; BMD, bone mineral density; CTx, C-terminal telopeptide; ITT, intention-to-treat; P1NP, procollagen type I *N*-terminal propeptide; PTH, parathyroid hormone; vBMD, volumetric bone mineral density.

## 5. The application of probiotics in the prevention and treatment of OP

**Figure 5.**
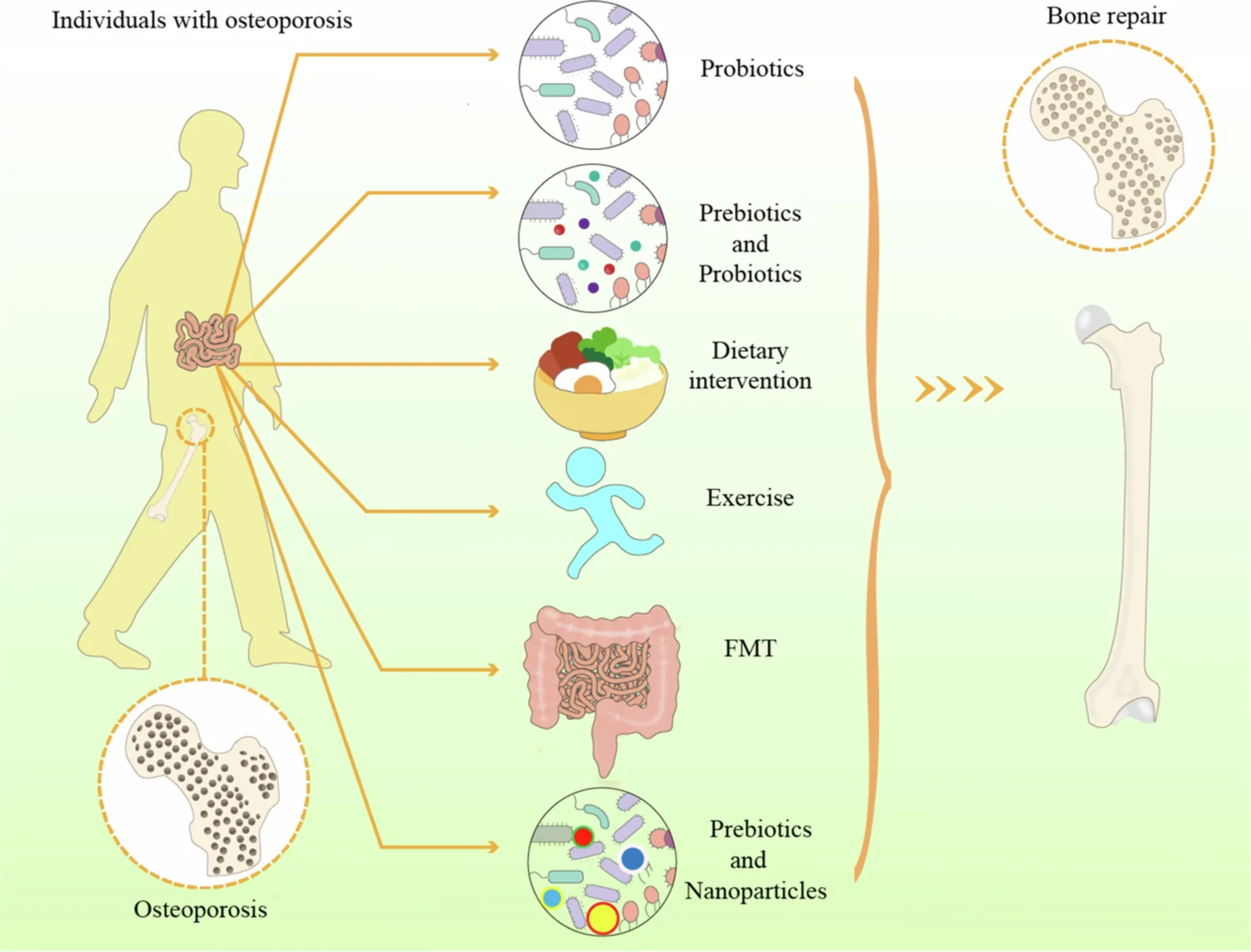
Clinical applications and therapeutic strategies targeting the gut–bone axis for the prevention and treatment of osteoporosis. Current microbiome-based interventions aimed at restoring bone metabolic balance include: direct probiotic supplementation, the combined application of prebiotics and probiotics (synbiotics) to enhance microbial colonization, dietary interventions (such as high-fiber and Mediterranean diets), targeted exercise to reshape the gut microbiota structure, fecal microbiota transplantation (FMT) to rebuild a healthy microbial ecosystem, and the innovative combined application of probiotics with nanoparticles to improve targeted delivery, survival rates, and colonization efficiency in the gastrointestinal tract.

Current clinical treatments primarily involve pharmacological interventions and nutritional supplementation but are associated with notable limitations. Long-term use of bisphosphonates can induce osteonecrosis of the jaw or atypical femoral fractures^193^; bone-forming agents (such as teriparatide) carry a risk of osteosarcoma; combined calcium and vitamin D supplementation provides only a modest 3%-5% increase in BMD for patients with moderate to severe OP. Calcium supplements may cause hypercalcemia, and estrogen replacement therapy may increase breast cancer risk.^194^ These drawbacks underscore the urgent need to develop novel therapeutic strategies with potent efficacy, high safety, and convenient administration. Based on our current understanding of how probiotics regulate bone metabolism through the gut‒bone axis, clinical applications of microbiome-based therapies include probiotics, the combined application of probiotics and prebiotics, dietary interventions, exercise, fecal microbiota transplantation (FMT), and the combined application of probiotics and nanoparticles (Figure 5).

### 5.1. Probiotic

Probiotic therapy is generally regarded as a relatively safe emerging treatment modality with a favorable side-effect profile. Its application in the medical field is increasingly widespread, especially for common health conditions. The frequency of probiotic use is steadily increasing.^195^ Particularly in the prevention and treatment of metabolic diseases such as osteoporosis, the primary focus of this review, probiotic therapies, hold broad application prospects. Probiotics may improve bone metabolism through multiple mechanisms, including enhancing calcium absorption, reducing inflammatory responses, and promoting bone formation. Both animal experiments and human studies have shown that probiotics can have a positive impact on bone health parameters, such as reducing osteoclast generation, lowering the expression of bone resorption markers, promoting the activity of osteoblasts, reducing osteocyte apoptosis, and increasing BMD and the expression of OPG.^196^ By ingesting sufficient amounts of probiotics, intestinal health can be effectively restored, thereby having a positive impact on bone health. Behera Jyotirmaya et al. found that *Lactobacillus reuteri* can reduce the expression of the inflammatory cytokine TNF-α in the jejunum and ileum and significantly increase trabecular density and trabecular number in the femurs and vertebrae of experimental mice.^197^ Therefore, we believe that probiotics can be powerful adjuvant to existing therapies for the prevention and management of OP.

### 5.2. Combined application of prebiotics and probiotics

The most widely recognized definition of prebiotics is formulated by the International Scientific Association for Probiotics and Prebiotics (ISAPP) in 2017: “A substrate that is selectively utilized by host microorganisms conferring health benefit.”^198^ Prebiotics are considered functional foods that are beneficial to health and are widely used in people's daily diet. There is a synergistic effect between prebiotics and probiotics. Probiotics need nutrients during their colonization and growth in the intestine, and prebiotics, as their “food,” can specifically provide nutrients for beneficial bacteria and promote their proliferation. At the same time, the activities of probiotics can improve the intestinal microenvironment, creating more favorable conditions for the further action of prebiotics. These components promote each other and jointly enhance the regulation of intestinal microecology. The combined application of prebiotics and probiotics in maintaining intestinal health and other related fields is of great significance.

Prebiotics include a large class of indigestible oligosaccharides composed of short-chain sugars, among which the most common are galacto – oligosaccharides (GOS), fructo – oligosaccharides (FOS), inulin, xylo – oligosaccharides (XOS), polydextrose, and lactulose. In the prebiotic group, a series of metabolizable food components, such as human milk, onions, garlic, and compounds from other vegetables, can also be included.^199^ Among them, fructo – oligosaccharides (FOS), galacto – oligosaccharides (GOS), inulin, and lactulose are currently the most extensively used prebiotics. Studies have shown that prebiotics can be ingested through eating relevant foods or other means to affect the gut microbiota, stimulate the growth of beneficial microorganisms and the production of beneficial metabolites, and thus maintain a balanced gut ecosystem.^200^ Synbiotics, which are mixtures composed of live microorganisms and substrates containing both probiotics and prebiotics, represent a combined therapeutic approach.^201^ Their application may enhance bone homeostasis.^202^

The combined application of probiotics and prebiotics is extensive. Relevant studies believe that it can affect the health of menopausal women by regulating the gastrointestinal microbiota, preventing OP during menopause and other related diseases, and beneficially influencing multiple metabolic risk factors^203^ (Table 4). The combined application of prebiotics and probiotics has potential advantages in regulating the balance of gut flora, enhancing immune function, preventing and treating diseases, etc. The gut microbiota can regulate the host's immune, metabolic, and endocrine systems through multiple pathways and plays a crucial role in preventing and treating OP. In addition, using probiotics and prebiotics to change the composition of the host's gut microbiota can provide new directions for the prevention and treatment of gut microbiota-related diseases. However, most of the current relevant research focuses on the field of animal experiments, and large-scale clinical studies and evidence-based data are relatively scarce in this field. Given the limitations of the current research, more high-quality clinical studies are still needed to clarify issues such as their mechanisms of action, optimal combination schemes, and the safety of long-term use. With the continuous deepening of research, the combined application of prebiotics and probiotics is expected to provide new ideas and methods for intestinal health and the treatment of related diseases.

### 5.3. Dietary intervention

The composition and content of nutrients in different types of food significantly influence the composition and abundance of the gut microbiota, which in turn affects bone metabolism and regulates OP. Dietary patterns such as high-fat and high-sugar diets have been shown to impair gut function, affecting intestinal pH and SCFA production. These alterations are accompanied by decreased calcium absorption, enhanced osteoclast activity, and accelerated bone turnover, ultimately contributing to bone mass loss. Generally, a diet high in fiber and whole grains, and low in sugar and fat, is recommended for preventing OP. Vegetarian diets and the Mediterranean diet can enhance the diversity of the gut microbiota, promoting the growth of beneficial bacteria such as Bifidobacterium, Lactobacillus, and Prevotella. The enrichment of these beneficial bacteria is associated with increased SCFA production, suppression of osteoclastogenesis, and preservation of trabecular bone microarchitecture. In contrast, a Western diet can reduce gut microbiota diversity and increase the Firmicutes-to-Bacteroidetes ratio, which has been linked to elevated systemic inflammation, increased bone resorption markers, and reduced bone mineral density.^204^ A meta-analysis of observational studies comprising 13,209 participants found that total hip BMD and trochanteric BMD were positively correlated with adherence to the Mediterranean diet.^205^ These findings suggest that diet-induced modulation of the gut microbiota translates into measurable improvements in skeletal parameters rather than merely intestinal changes. A 6-month clinical trial demonstrated that daily supplementation with anthocyanin-rich blackcurrants modulated an increase in Ruminococcus 2, increased the BMD in postmenopausal women, and reduced the levels of the inflammatory cytokines IL-1β and IL-2. Therefore, a rational dietary pattern, based primarily on the principle of food diversification, is recommended. It involves consuming more vegetables, fruits, legumes and their products, fish, and seafood, while limiting the intake of red meat and saturated fatty acids. Such a rational dietary pattern also lays a solid foundation for shaping intestinal homeostasis, promoting the proliferation of beneficial bacteria, eliminating harmful bacteria, and maintaining the diversity of the gut microbiota. Through these mechanisms, dietary modulation of the gut microbiota contributes to the maintenance of bone mass, improvement of bone microstructure, and reduction of osteoporosis risk, which is crucial for the prevention and treatment of OP.^8^

### 5.4. Exercise

Exercise is currently one of the most common, reliable, and cost-effective interventions for preventing and treating OP. The close association between the gut microbiota and bone metabolism means that exercise can alter the structure, composition, and abundance of the gut microbiota, thereby influencing bone metabolism through the microbiota and its metabolites.^206^ When engaging in different types of exercise, distinct changes occur in the composition and abundance of the gut microbiota. These changes affect nutrient absorption, energy allocation, immune function, inflammation regulation, and intestinal mucosal barrier function, thereby modulating the onset and progression of OP and other bone diseases.^207^ The relationship between exercise intervention and the gut microbiota is complex and depends on several factors, such as exercise type, intensity, duration, frequency, and the surrounding environment. The relative abundance of Verrucomicrobia, Verrucomicrobiaceae, Akkermansia, and Dorea in the feces of sedentary, overweight women increased after 6 weeks of moderate-to-low intensity dynamic bicycle training.^208^ Researchers conducted a 14-week intervention study on mice fed either a high-fat diet (HFD) or a low-fat diet (LFD). The mice were divided into a voluntary exercise group and a sedentary group. The results showed that an HFD led to reduced trabeculae in the vertebrae and tibia, increased BM fat, and GM dysbiosis in the mice. However, exercise mitigated the HFD-induced GM dysbiosis. It was observed that exercise reduced the Firmicutes-to-Bacteroidetes ratio, lowered the levels of Clostridia and Methanobacteriaceae, and increased the abundance of Actinobacteria members (specifically Bifidobacteriaceae). These alterations are beneficial for reshaping the structure and abundance of the gut microbiota and improving OP.^209^ Furthermore, exercise can prevent gut dysbiosis associated with energy storage while increasing BMD.

### 5.5. FMT

In FMT treatment, feces from healthy humans or animals are processed to obtain beneficial microbial communities for transplantation into the recipient's gastrointestinal tract. The therapeutic effects of FMT for OP primarily involve the modulation of the intestinal barrier, the gut microbiota structure, and immune responses. FMT inhibits excessive osteoclastogenesis and prevents OVX-induced bone loss. It enhances the expression of ZO-1 and suppresses the release of TNF-*α* and IL-1β. Furthermore, FMT increases the abundance of Bacteroidetes, reduces the abundance of Mucoromycota, and elevates fecal SCFAs.^166^ Research on FMT therapy for OP is relatively limited and has focused primarily on animal experiments. More clinical trials are needed to determine the mechanisms involved and improve therapeutic efficacy.

### 5.6. The combined application of probiotics and nanoparticles

#### 5.6.1. Nano protection strategies for enhancing the survival rate and intestinal colonization ability of probiotics

The therapeutic potential of probiotics is constrained by multiple challenges, including their low survival rate in the gastrointestinal tract, imprecise delivery, and limited mechanistic scope. Consequently, their efficacy in clinical trials remains suboptimal, hindering broader development and application. The integration of nanoparticles – enabling the synergistic use of nanoparticle-probiotic hybrids – offers a promising strategy to overcome these limitations. In recent years, single-cell nano-encapsulation has emerged as a transformative approach. First, this technique establishes a nanoscale protective barrier around each viable probiotic cell.^210^ ^,^ ^211^ Notably, dual-layer chitosan/alginic acid encapsulation ((CHI/ALG)₂) has been shown to significantly enhance probiotic survival under harsh gastric acidity and bile salt exposure.^212^ Second, nanoencapsulation actively promotes intestinal adhesion and colonization. Coatings fabricated from mucosal-adhesive materials – such as positively charged chitosan^213^ or biomimetic biofilm components^214^ – strengthen non-specific interactions between probiotics and the intestinal mucus layer via electrostatic forces or biorecognition mechanisms. Animal studies have demonstrated that nanoencapsulated probiotics exhibit orders of magnitude improvement in both intestinal residence time and colonization duration.^215^ ^,^ ^216^ Furthermore, the single-cell encapsulation platform confers programmable functionalities that extend beyond the native capabilities of probiotics. By leveraging the biotin-avidin system, antimicrobial peptides, anti-inflammatory agents, or immunomodulators can be precisely immobilized on the probiotic surface. This dual-functional design enables engineered probiotics to maintain their intrinsic roles in microbiota modulation and pathogen inhibition while simultaneously delivering therapeutic payloads locally at the site of colonization.^216^

#### 5.6.2. Development of a multifunctional nano packaging platform for reconstructing the pathological microenvironment

The development of multifunctional nanoplatforms for reconstructing the pathological microenvironment represents one of the most innovative and promising frontiers in biomedical research, aiming to engineer intelligent nano systems capable of simultaneously modulating multiple pathological pathways. A representative example is metal-poly DNA nanoparticles (Ca-polyCpG MDNs), which function as a multifunctional platform with demonstrated multi-target regulatory capacity. Upon systemic administration, these nanoparticles selectively accumulate in regions of active bone resorption. Evidence indicates that Ca-polyCpG MDNs effectively suppress osteoclast differentiation at disease sites.^217^ Meanwhile, specific probiotic strains – such as *Lactobacillus rhamnosus* GG and *Lactiplantibacillus plantarum* – have been shown to modulate systemic immune homeostasis.^217^ Consequently, the combination of probiotic therapy with immunomodulatory nanoparticles like Ca-polyCpG MDNs enables synergistic therapeutic effects across complementary physiological levels: probiotics primarily act within the gut microenvironment, promoting a systemic anti-inflammatory state via the “gut–bone axis”; in parallel, the nanoparticles directly target skeletal lesion sites, enabling precise modulation of the local immune microenvironment.

**Table 4.** Literature on the results and mechanisms of prebiotic treatment of osteoporosis in animal models.

Animal model	Prebiotic/intervention	Treatment dose	Treatment duration	Skeletal outcomes	Immune effects	Gut/barrier effects	Microbiota modulation	Mechanism/conclusion	Ref.
Natural aging model (14-month-old female C57BL/6J mice)	2′-Fucosyllactose (2′-FL)	500 mg/kg body weight by gavage (200 μL/day)	12 weeks	BMD ↑; BV ↑; BV/TV ↑; Tb.N ↑; Tb.Th ↑; Tb.Sp ↓	M1-polarized macrophages ↓; pro-inflammatory signaling/cytokines ↓	Colonic inflammation ↓; ZO-1 and Occludin restored	Diversity restored; *Bifidobacterium* ↑; Prevotellaceae ↑; *Akkermansia* ↑; *Stenotrophomonas* ↓	The suppression of M1 macrophage polarization by TLR4-mediated MyD88/NF-κB signals	[218]
Calcium-deficient model (male ICR mice)	Konjac oligosaccharides (KOS)	2%, 4%, or 8% KOS replacing corn starch in diet	8 weeks	Negative Ca balance alleviated; Tb.N ↑; cortical thickness ↑; skeletal mechanical strength ↑	Not assessed	Gut environment and cecal indices improved	*Lactobacillus*, *Bifidobacterium*, *Mucispirillum*, *Alistipes* and unidentified *Clostridia* associated with increased bone mass	KOS affected the Ca balance status and bone mass through gut microbiota and metabolic regulation	[219]
OVX model (female Sprague-Dawley rats; NTac:SD)	Inulin and lactose	Inulin 5% or lactose 0.5% in the diet with calcium supplementation	6 weeks	Minor beneficial effects on spine BMD/BMC; lactose significantly increased spine BMD/BMC in calcium-supplemented groups	Not assessed	Inulin: SCFAs ↑, intestinal pH ↓, Trpv6/Aqp8 ↑; lactose: jejunal pH ↓	Inulin: *Allobaculum* ↑ and *Bifidobacterium* ↑; lactose: no marked microbiota effect	SCFAs↑ pH value↓ Trpv6 and Aqp8 in the lower gastrointestinal tract (lower GI) ↑	[220]
Growing rat model (4-week-old male Sprague-Dawley rats)	Galactooligosaccharides (GOS)	0%, 2%, 4%, 6%, or 8% GOS in diet	8 weeks	Distal femur total/trabecular vBMD ↑; proximal tibia vBMD ↑; femur/tibia breaking strength ↑; Ca/Mg absorption and retention ↑	Not assessed	Cecal pH ↓; cecal wall/content weight ↑	*Bifidobacteria* ↑; microbial community structure altered	Cecal pH↓ Cecal wall weight and content weight↑ Gut microbiota community structure proportion of bifidobacteria↑	[221]
OVX model (female rats)	Milk calcium-fortified yogurt ± inulin	Milk calcium-fortified yogurt, with or without inulin	6 weeks	Yogurt increased spine BMD/BMC; no additive bone benefit from inulin	Not assessed	Inulin markedly increased SCFAs and lowered gut pH	Yogurt: lactobacilli ↑, Clostridiaceae ↓; yogurt + inulin: *Bifidobacterium pseudolongum*, *Turicibacter*, *Blautia* and *Allobaculum* ↑	Lactobacilli↑ Clostridiaceae ↓ pH in the proximal part of the intestine↓	[222]
OVX model (female Wistar rats)	Standardized aqueous extract of *Anoectochilus formosanus* (SAEAF)	200 or 400 mg/kg/day orally	12 weeks	BMD/BMC and biomechanical strength preserved; trabecular bone volume ↑	Not assessed	Ca absorption/retention ↑; caecal pH ↓; calbindin-D9k mRNA ↑; free Ca ↑; SCFAs ↑	*Bifidobacteria* ↑	Microbial fermentation products of prebiotics are responsible for an increase in Ca absorption in the large intestine.	[223]
OVX model (8-week-old female ddY mice)	Resistant starch (HAS/AH-HAS)	20% HAS or 20% AH-HAS diet (6.8% or 12% resistant starch)	6 weeks	OVX-induced femoral bone loss attenuated; whole/proximal femur BMD preserved	Colon IL-10 mRNA ↑ (trend); bone-marrow RANKL ↓ and IL-7R ↓	Cecal fermentation increased; pH ↓	*Bifidobacterium* spp. ↑	AH-HAS-containing diet accelerated fermentation in the caecum Bifidobacterium spp↑ IL-10 ↑ RANKL↓ IL-7R ↓	[223]
Senescence-accelerated model (male SAMP6 mice)	Fructooligosaccharide (FOS)/glucomannan (GM)	5% FOS or 5% GM in diet	31 weeks	Femoral Ca content ↑ with FOS; urinary deoxypyridinoline ↓ with FOS and GM	hs-CRP ↓	Not assessed	FOS: *Lactobacillus* ↑ and *Bacteroides* ↑; GM: *Clostridium* ↑	Mineral absorption in the gastrointestinal tract↑ Inflammation↓	[224]

Abbreviations: ↑, increase; ↓, decrease; BMC, bone mineral content; BMD, bone mineral density; BV, bone volume; BV/TV, bone volume fraction; hs-CRP, high-sensitivity C-reactive protein; OVX, ovariectomy; SCFAs, short-chain fatty acids; Tb.N, trabecular number; Tb.Sp, trabecular separation; Tb.Th, trabecular thickness; vBMD, volumetric bone mineral density.

## 6. Discussion and further directions

### 6.1. Research value and future directions of the gut–bone axis in osteoporosis

OP is characterized by decreased BMD and an increased risk of fragility fractures, particularly in the aging population. Despite the availability of anti-resorptive and anabolic therapies, current treatments are limited by suboptimal efficacy, adverse effects, and poor long-term compliance. In this context, the gut–bone axis has emerged as a novel and promising framework for understanding OP pathogenesis and developing alternative preventive and therapeutic strategies.

Accumulating evidence demonstrates that the gut microbiota plays a critical role in bone homeostasis by regulating nutrient absorption, immune responses, endocrine signaling, intestinal barrier integrity, and microbial metabolite production. These mechanisms collectively influence osteoclast–osteoblast coupling and contribute to the development of postmenopausal osteoporosis (PMO). Importantly, this review extends previous gut–bone axis studies by emphasizing the integrative role of intestinal biomechanics and host–microbiome interactions, thereby providing a more comprehensive mechanistic perspective.

From a translational standpoint, microbiome-targeted interventions have been increasingly evaluated in both experimental and clinical settings. Animal studies have consistently shown that specific probiotic strains can prevent bone loss and improve BMD under estrogen-deficient or inflammatory conditions. In clinical trials, supplementation with probiotics – particularly strains belonging to *Lactobacillus* and *Bifidobacterium* – has demonstrated modest but significant improvements in BMD and bone turnover markers in postmenopausal women. However, these trials vary considerably in study design, strain selection, dosage, and duration, highlighting the need for more standardized and large-scale clinical validation. Compared with previous reviews, this manuscript provides a structured analysis of these clinical studies, critically evaluating their strengths, limitations, and translational relevance.

Emerging strategies such as FMT have shown potential in restoring the gut microbial balance and improving metabolic and immune functions, although direct clinical evidence for OP remains limited. Future research should focus on identifying bone-protective microbial signatures, elucidating strain-specific mechanisms, and validating their efficacy and safety in rigorously designed clinical trials. Notably, there are currently no microbiota-based therapies approved specifically for OP, underscoring a significant gap between experimental findings and clinical application.

In addition, combined interventions involving probiotics and prebiotics may offer synergistic benefits by enhancing microbial colonization and metabolite production. Nevertheless, host-specific differences in gut microbiota composition may lead to heterogeneous responses, emphasizing the importance of personalized approaches. With advances in microbiome profiling and precision medicine, individualized microbiota-targeted therapies may become feasible, offering more effective and tailored treatment options for patients with OP.

### 6.2. Research value and future directions of specific probiotics

Although a wide range of probiotics has been reported to influence bone metabolism via the gut–bone axis, not all bacterial taxa hold equal translational or mechanistic value for future osteoporosis research. Current evidence suggests that genera represented by *Lactobacillus* and *Bifidobacterium* remain the most promising candidates for further investigation and clinical application, owing to their relatively high safety profiles, mechanistic diversity, and robust experimental and clinical evidence.^225^ These probiotics exert osteoprotective effects through multiple interconnected pathways, including immunomodulation, microbial metabolite production, intestinal barrier repair, and endocrine regulation.^226^

One major reason these genera are particularly valuable lies in their capacity to modulate osteoimmune interactions. Osteoporosis, especially postmenopausal osteoporosis, is increasingly recognized as an immune-mediated disorder characterized by excessive osteoclast activation driven by inflammatory cytokines. Experimental studies have shown that *Lactobacillus rhamnosus* GG can ameliorate osteoporosis by restoring the Th17/Treg balance and reshaping the gut microbiota composition in ovariectomized rat models.^35^ Consistently, *Lactobacillus rhamnosus* has been reported to suppress pro-osteoclastogenic cytokines such as IL-6, IL-17, and TNF-*α* while enhancing anti-osteoclastogenic cytokines including IL-4, IL-10, and IFN-*γ*, thereby protecting bone mass under inflammatory conditions.^38^ In addition, *Bifidobacterium longum* has been shown to enhance the anti-osteoclastogenic activity of regulatory B cells (Bregs), significantly alleviating ovariectomy-induced bone loss.^188^ These findings highlight the particular value of immune-targeted probiotics for future osteoporosis prevention strategies. Beyond immune regulation, metabolite-mediated pathways further support the research value of specific probiotics. Certain *Bifidobacterium* and *Lactobacillus* strains can promote the production of SCFAs, such as butyrate and propionate, which are increasingly recognized as key mediators of gut–bone communication. Clinical evidence indicates that *Bifidobacterium* lactis Probio-M8 improves bone metabolism in postmenopausal osteoporotic patients by modulating the gut microbiota structure and enhancing SCFA production.^190^ SCFAs have been shown to inhibit osteoclast differentiation, promote osteoblast activity, and regulate T cell-dependent bone remodeling.^227^ ^,^ ^228^ In animal models, *Lactobacillus rhamnosus*-derived butyrate promotes bone formation through Treg-mediated Wnt10b signaling.^58^ In addition, SCFAs can directly regulate osteoblast and osteoclast function via histone deacetylase (HDAC) inhibition while indirectly exerting anti-inflammatory effects.^228^ Emerging evidence further suggests that *Bifidobacterium longum* may alleviate osteoporosis by increasing serum vitamin D metabolite levels, providing a novel mechanistic link between the gut microbiota and the endocrine regulation of bone metabolism.^229^

Another critical but often overlooked aspect is intestinal barrier integrity. Increased intestinal permeability is commonly observed in osteoporosis and facilitates the translocation of LPS, thereby exacerbating systemic inflammation and bone resorption. Probiotic lactobacilli have been shown to prevent post-antibiotic bone loss by reducing gut dysbiosis and preserving intestinal barrier function through decreased permeability and protection of tight junction proteins.^138^ Moreover, mixed probiotic formulations containing *Bifidobacterium* and *Lactobacillus* can repair intestinal barrier damage in corticosteroid-induced osteoporosis models, reduce LPS translocation, and indirectly protect bones by attenuating systemic inflammation.^230^ In a rat model, *Bifidobacterium animalis* subsp*. lactis* BL-99 significantly reduced inflammatory cytokine levels while upregulating tight junction-related proteins (Claudin-1, MUC2, ZO-1, and Occludin), ultimately improving bone microarchitectural parameters, such as BV/TV and trabecular thickness.^230^ Importantly, accumulating evidence indicates that probiotic effects on bone are highly strain-specific rather than genus-dependent. For instance, *Lactobacillus reuteri* ATCC PTA 6475 has demonstrated clinical efficacy in reducing bone loss in elderly women,^188^ whereas specific subspecies of *B. longum* can upregulate osteogenic genes such as SPARC and BMP-2, thereby promoting bone formation.^231^ Meta-analyses further suggest that *Lactobacillus* species often exert stronger effects than *Bifidobacterium* in improving BMD and BV/TV, and that multi-strain formulations may produce synergistic benefits.^180^ Taken together, these findings underscore the importance of prioritizing strain-level screening and functional validation in future probiotic research. Integrating host-specific gut microbiota characteristics may enable the development of precision probiotic interventions tailored to individual osteoporosis phenotypes. Such approaches have the potential to shift probiotics from adjunctive supplements to core, safe, and cost-effective therapeutic strategies for osteoporosis prevention and treatment.

Currently, research on specific application protocols for probiotics, prebiotics, and synbiotics remains limited, with a notable lack of clinically meaningful guidelines for combination therapies and personalized treatment strategies. Furthermore, in practical clinical applications, there is an absence of guiding studies on how to precisely extract, purify, or supplement required prebiotic or synbiotic preparations from patients’ complex dietary structure. Most trials employ a “one-size-fits-all” fixed formulation, neglecting the inherent dynamic homeostasis within the host. Consequently, there is an urgent need to transition from rigid experimental formulas to personalized intervention studies. Additionally, stratified research examining the dose‒response relationship is highly insufficient, and comparative studies on probiotics, prebiotics, and synbiotics with varying formulation ratios remain inadequate. This ultimately hinders their evidence-based clinical translation. To address these critical limitations, future research should focus on strain-specific screening, the personalized adaptation of standardized dosage regimens, and the development of individualized microbiome treatment protocols.

In short, probiotics play an important role in the occurrence and development of OP through the bone‒gut axis. A deeper understanding of this relationship will help develop new prevention and treatment methods and bring hope to millions of people with OP around the world.
